# Intermittent gas–liquid rearrangement controls the mixing of gases in porous media

**DOI:** 10.1073/pnas.2612721123

**Published:** 2026-11-05

**Authors:** Samuel J. Jackson, Sharon Ellman, Gülce Kalyoncu, Tanguy Le Borgne, Tom Bultreys

**Affiliations:** ^a^ Energy Research Unit, Commonwealth Scientific and Industrial Research Organisation (CSIRO), Clayton, VIC 3168, Australia; ^b^ Department of Geology, Centre for X-ray Tomography, Ghent University, Ghent 9000, Belgium; ^c^ Géosciences Rennes, Université de Rennes, CNRS, Unité Mixte de Recherche, Rennes 35000, France; ^d^ The Njord Centre, Department of Geosciences, University of Oslo, Oslo 0316, Norway; ^e^ Institute of Environmental Assessment and Water Research (IDAEA), Spanish National Research Council (CSIC), Barcelona 08034, Spain

**Keywords:** multiphase flow, porous media, multicomponent, gas, mixing

## Abstract

Gas mixing in porous media governs important environmental and industrial applications in which multiple gas components share the available pore space with liquid water, simultaneously undergoing gas mixing and gas–liquid redistribution. Currently, this process is represented by macroscopic models that ignore pore scale dynamics. We demonstrate that this is not generally valid, and can lead to large errors in the mixing rate. We use 4D X-ray imaging to investigate nitrogen injection into a porous sandstone saturated with krypton and water, jointly imaging the gas mixture composition evolution and multiphase flow of gas and water. In contrast to the smooth, continuous representation of the conventional advection–diffusion model, our results show that gas mixing evolves through a sequence of distinct regimes controlled by gas connectivity. Initially, immiscible gas–water displacement generates gas composition variance as the different gases occupy neighboring pores separated by water. As gas connectivity increases, intermittent gas–liquid interfacial rearrangements rapidly connect regions of contrasting gas composition, driving a fast decay in composition variance. Once connectivity stabilizes, mixing is controlled by a confined diffusion process within the quasi-static gas network. At later times, further mixing is driven slowly by multicomponent Ostwald ripening, whereby trapped gas ganglia exchange solutes through the surrounding liquid phase. These findings provide insights into gas mixing in porous media, suggesting concepts for upscaling the role of pore scale multiphase flow in gas mixing in the subsurface.

## Significance

Understanding how gases mix with each other in a porous medium is important in a range of natural and engineering processes, from oxygen perfusing in soils to hydrogen blending with residual natural gas in underground hydrogen storage reservoirs. In many such cases, the pores also contain liquid water that flows and rearranges in interaction with the gases. We show that this plays a pertinent and so far elusive role in the rate at which gases mix. Using an innovative X-ray imaging method, our experiments reveal that dynamic gas–liquid rearrangements, occurring intermittently in bursts, act as a gatekeeper for diffusive gas mixing in the pore space. This process may play a key role in important applications such as underground hydrogen storage.

Gas transport in partially saturated porous media is a key process governing many natural and engineered processes such as gas exchange between the vadose zone and the surface, containment of volatile pollutant transport (1, 2), parasitic flow in redox batteries (3, 4), the hydration reaction in fuel cells (5), and underground storage of hydrogen and carbon dioxide (6). Of particular interest in this work is underground hydrogen storage (UHS), where compressed hydrogen gas is stored in deep underground porous rock layers such as depleted natural gas fields and saline aquifers. This technology is being explored globally as a means to store large-scale excess renewable energy and alleviate fluctuations in energy demand (7). After injection, the hydrogen gas mixes with residual natural gas or cushion gases (preinjected gases to maintain pressure e.g. carbon dioxide, nitrogen, methane), in the presence of saline groundwater (brine) (6). This mixing process has important implications for recovery and chemical reactivity of the stored gas, and hence for economic feasibility of this technology. It is thus paramount to have reliable quantitative models of how gases mix with each other, typically based on numerical solutions of the advection-dispersion equation (8).

The description of mixing is complicated by the fact that the porous medium in the cases above is typically wetted by a liquid, present in the pores along with the gas components. The prevailing condition in most applications, and indeed during UHS and carbon capture & storage (CCS), is simultaneous coevolution of both the gas composition and the liquid saturation. In continuum-scale modeling, the presence of the liquid phase is commonly taken into account by reducing the effective gas diffusivity and increasing the interstitial fluid velocity (9). The former is typically expressed using a scaling constant (tortuosity) which depends solely on the momentary saturation averaged over a meter-scale grid block. The pore–scale interaction of saturation dynamics and mixing is thus considered to be negligible in these simulations.

Despite the assumptions in the continuum approach, there is increasing evidence that pore–scale fluctuations may not simply average out (10). At the microscopic scale of the pores, gas–liquid menisci undergo abrupt motions when the saturation changes. This continuously alters the local flow velocity and the connectivity of the fluids in the pore network while the gas composition evolves (10). It is currently unclear if and how this pore scale coupling impacts the effective mixing, despite extensive work on solute transport in partially saturated porous media. Experimental and theoretical studies on this topic have focused on liquid phase solute transport as opposed to gas-phase element transport (11–15). Recent advances highlight that fluid motions can cause fluctuations in pore scale velocity fields (16–18), which could potentially enhance dispersion in partially saturated media. Nevertheless, most experimental work has been focused on specific situations where the fluid distribution remains immobile while the advective-diffusive transport takes place (11, 13, 14, 16). In these cases, the existence of multiple fluid phases generally leads to non-Fickian solute transport, with regions of flow channeling and stagnation exhibiting a broad range of breakthrough times (14). However, there are currently no experimental observations of unsaturated multicomponent gas mixing in porous media, particularly when gas composition and saturation coevolve.

In this work, we utilize a methodology based on laboratory-based micro-CT with carefully selected fluids (KI-doped water, krypton gas, and nitrogen gas) to enable 4D imaging of binary gas mixing and liquid transport in a porous medium. Thus, we capture the simultaneous coevolution of gas composition and saturation at the pore scale, under (near) subsurface conditions in a partially saturated sandstone rock sample. The achieved spatial and temporal resolutions enable us to track gas–brine interfaces and gas–gas mixing dynamics as the saturation front displaces through the sample. The experiments reveal key pore scale dynamics that break down the assumption of the classical Darcy scale advection–diffusion representation, with important implications for the aforementioned applications, and in particular for UHS.

## 1. Results

**Fig. 1.**
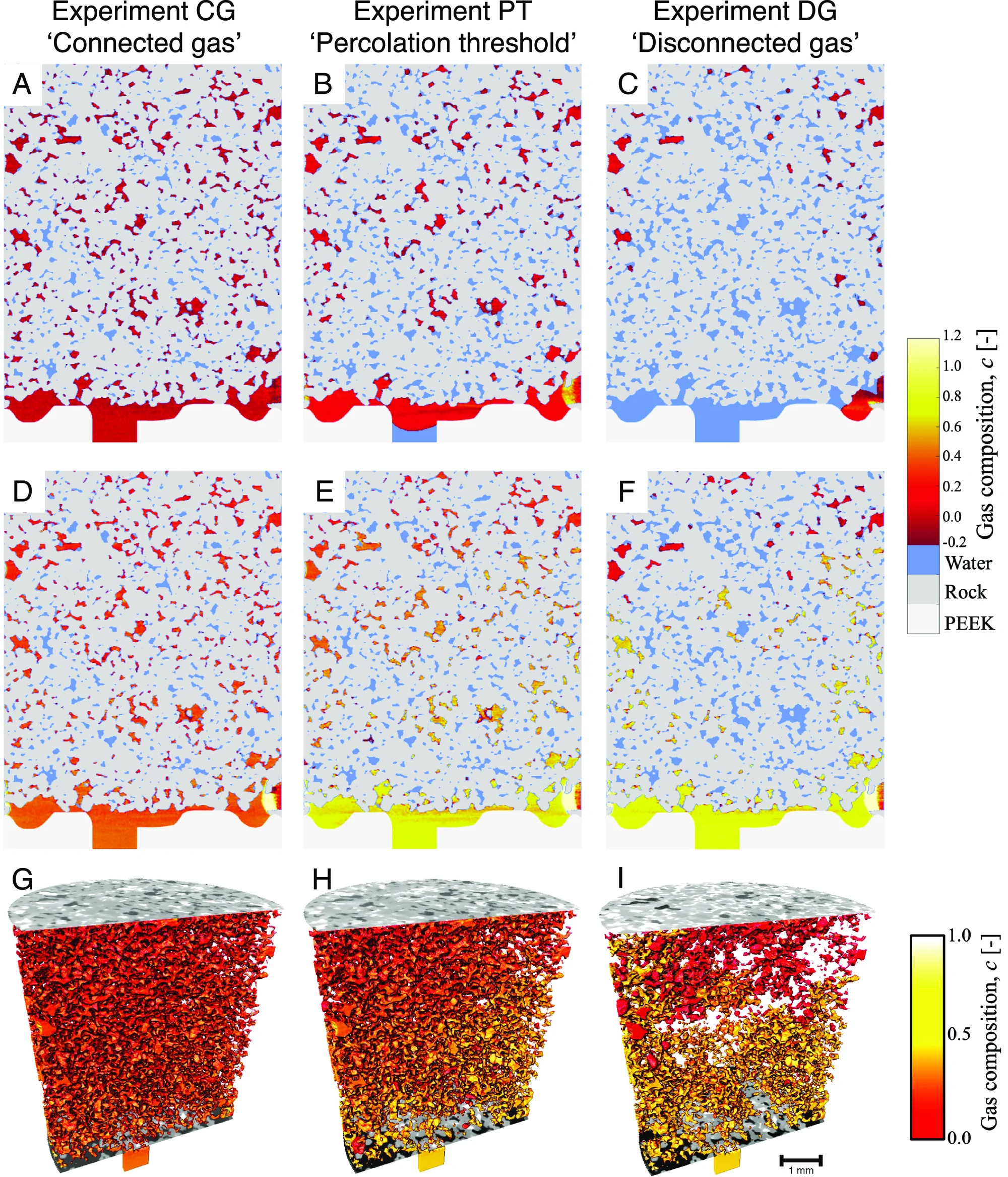
Micro-CT based maps of the binary gas composition show the impact of gas–liquid redistribution on gas mixing between krypton (c=0) and nitrogen (c=1) in the three experiments. (*A*–*C*) The initial water-krypton distribution differs between the experiments, shown here by 2D cross-section images along the injection direction. (*D*–*F*) Cross-sections at the time of maximum gas composition variance show that experiment CG exhibits a smooth gas composition gradient along the injection axis, while PT and DG have much sharper gradients. The associated video shows that sharp gradients are abruptly destroyed once enough nitrogen has entered the sample. (*G*–*I*) Corresponding 3D renderings show that sharp gas composition contrasts are associated with compartmentalized gas clusters. The associated videos show that sudden mixing occurs when clusters of the different gases connect, and that trapped gas bubbles slowly change composition. All videos are available in the *SI Appendix*, Movies S1–S3 show the 2D cross-sections, Movies S4–S6 show the 3D renderings.

Our experiments are designed to mimic hydrogen injection in depleted natural gas fields, but have general implications for gas transport in unsaturated porous media. We perform three unsteady gas-water flow experiments in a cylindrical Bentheimer sandstone sample with diameter 6 mm and length 26.2 mm. We use pressurized krypton and nitrogen gas as analogues for natural gas and hydrogen, respectively, due to their comparable relative fluid properties but higher X-ray attenuation contrast. In all three flow experiments, the sample is initially saturated with water doped with potassium iodide (20% wt. KI in deionized water). Following this, three different initialization procedures are used to set up different krypton saturations and connectivities (Fig. 1), which we show to have an important impact on the dynamics. Table 1 details the krypton and subsequent water injection rates used to initialise the sample, with water injected only in the latter two experiments.

**Table 1.** Experimental setup & initialization summary

Experiment name	CG	PT	DG
Krypton injection rate [ml/min]	0.03	0.45	0.03
Water injection rate [ml/min]	0.00	0.03	0.03
Initial average liquid saturation, $\bar{S_{l}}$ [-]	0.218	0.501	0.804
Initial gas connectivity χ [-]	0.5261	0.0578	−0.0690
Γ [-]	0.9836	0.5952	0.0064

The first experiment starts from a high gas saturation, with a well-connected cluster of gas in the pores. The second experiment has a lower initial gas saturation close to the percolation threshold, the critical saturation at which a large sample-spanning gas cluster can exist. Finally, the third experiment has the lowest gas saturation, with disconnected clusters of gas spread through the pores. We will refer to these experiments further as “Connected Gas” (CG), “Percolation Threshold” (PT) and “Disconnected Gas” (DG). These initial saturation conditions are of interest to, for example, hydrogen storage in repurposed natural gas fields, where a lower gas saturation represents an enhanced level of natural gas depletion. The setup and initialisation procedure are further detailed in *Material and Methods* and the *SI Appendix*.

Following the initialization procedure, nitrogen is injected in a laminar (Reynolds number, $Re\approx 10^{-3}$) and capillary dominated flow regime (pore–scale capillary number $Ca\approx 10^{-9}$, macroscopic capillary number, $N_{c}\approx 10^{-1}$), simultaneously displacing water and krypton gas. At this slow injection rate, nitrogen–krypton mixing is expected to fall in the restricted diffusion regime at the pore scale (local Péclet number, $Pe_{l}\approx 10^{-3}$) and in the transition regime toward advective dispersion at the sample scale (global Péclet number, $Pe_{g}\approx 1$) (19)—see *Materials and Methods* for full details on characteristic scalings.

To quantify the fluid redistribution and gas composition in the 3D pore space, time series of micro-CT scans are acquired during fluid injection, with a time resolution of 40 s per 3D scan, and a voxel size of 12.6 μm. The images capture the part of the sample near the fluid injection face. The micro-CT data is first analyzed by segmenting the three phases (rock, gas, liquid). Subsequently, for each gas voxel, the binary gas composition of krypton (c=0) versus nitrogen (c=1) is calculated, based on priorly recorded calibration images with known partial pressures of krypton in the sample. This enables us to quantify the evolution of the gas composition in specific pores for the experiments with different initial conditions, revealing distinct pore–scale mixing mechanisms. Fig. 1 shows 2D and 3D images/videos of the global time-lapse flow behavior. In the following analysis, we first discuss the pore scale mixing mechanisms, their late-time behavior, and finally show their impact on continuum-scale mixing dynamics.

### 1.1. Gas Connectivity Acts as a Gatekeeper for Pore Scale Mixing.

The experiments reveal markedly different mixing behavior depending on the gas–liquid distribution at the start of injection (Fig. 1). Experiment CG maintains a near-constant arrangement of gas and liquid throughout the measurements, with nearly all of the gas forming one large connected cluster. The gas composition changes smoothly, as expected in an advective–diffusive mixing scenario. In experiments PT and DG, on the other hand, water is displaced by the invading nitrogen gas, increasing the total amount of gas (nitrogen or krypton) in the pores. The 3D videos of these experiments (Movies S4–S6) show that on the whole, the connectivity between clusters of the defending and invading gas phases increases when this happens. Connection events between clusters of the different gases act as a gate keeper for the diffusive mixing occurring in the pores, with invasion events often occurring in bursts or avalanches, where many pores are reconnected near simultaneously. In some cases, defending gas clusters become or remain fully isolated, yet slowly change composition over time.

**Fig. 2.**
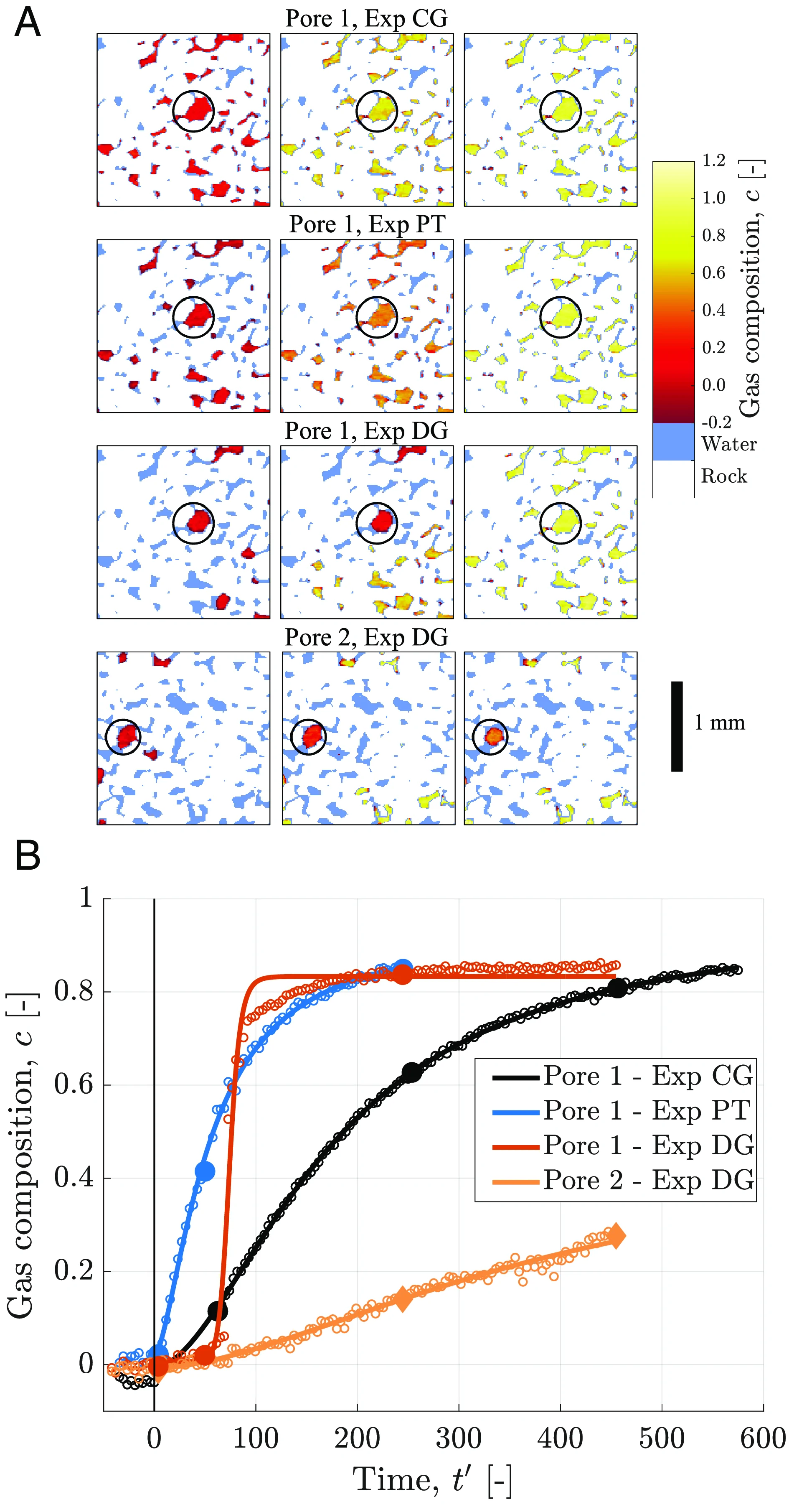
Snap-shots for two selected pores at three different times highlight the occurrence of different mixing mechanisms in the experiments. $t^{\prime }$ is the nondimensional time characterized by the pore scale advection time, see Eq. **5**. (*A*) Pore 1 undergoes smooth diffusive mixing in experiment CG, versus much more abrupt, intermittent mixing due to gas reconnection in experiment DG. Pore 2 shows a trapped gas bubble changing composition over time. The three snapshot times are highlighted by filled symbols in (*B*). Associated videos are available in Movies S7–S12. (*B*) Mean pore body gas composition in time for the pores in (*A*), with lines showing the logistic fit to the data.

To investigate the mixing mechanisms at the pore scale, we zoom in on the behavior of two selected pores in Fig. 2, highlighting both the mean pore body composition and the 3D spatial distribution in time. The behavior in “Pore 1” (Fig. 2*A*) illustrates the smooth, more gradual gas mixing during experiment CG compared to PT and DG. In experiment DG, krypton gas remains trapped in this pore for an extended time period as it is surrounded by water in neighboring pores. This effectively compartmentalizes the gas composition spatially. A rapid pore filling event then occurs across 2 to 3 time-steps for the pore body, mixing nearly pure nitrogen with the krypton, rapidly changing the gas composition (Fig. 2*B*).

**Fig. 3.**
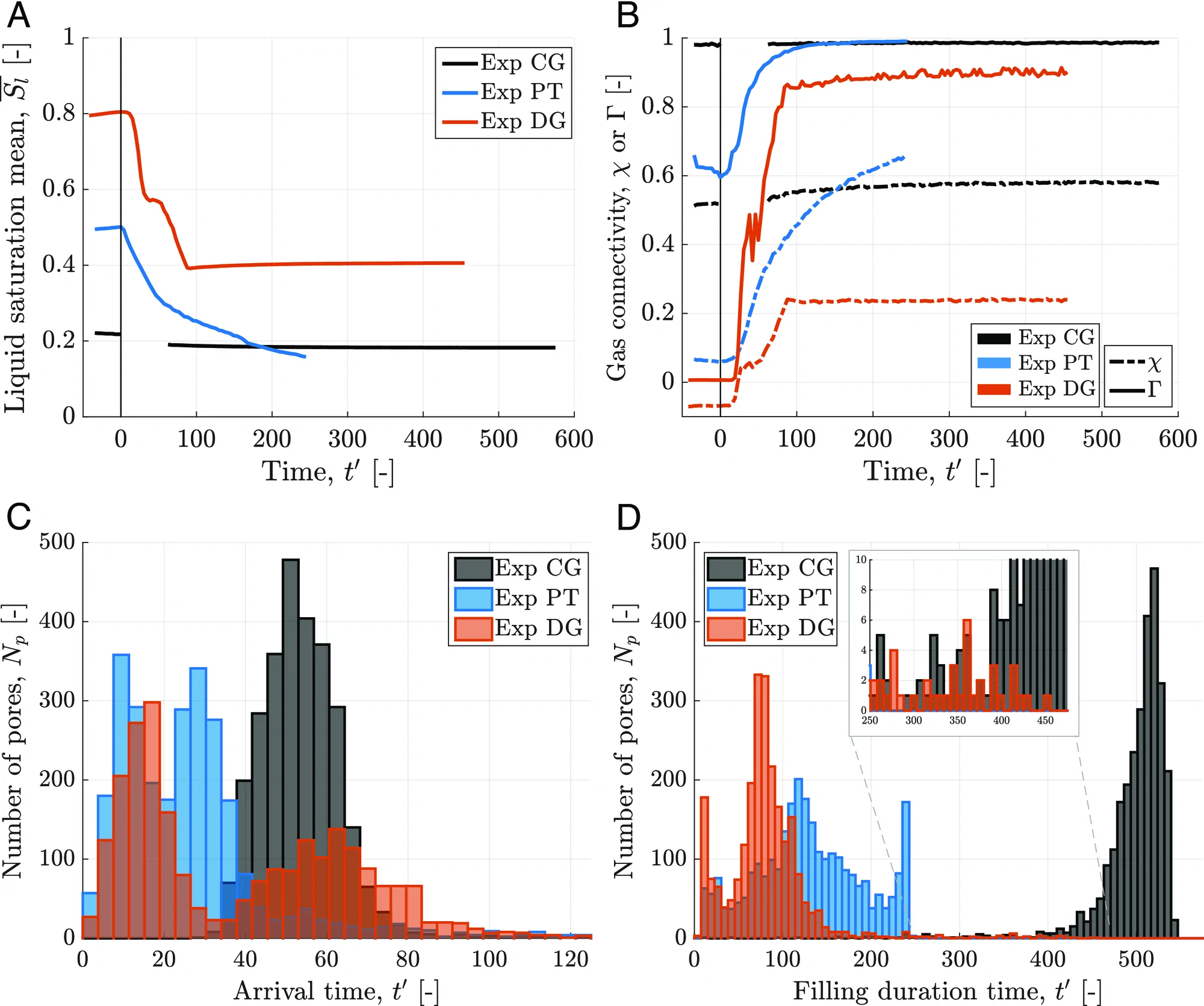
Pore scale statistics. (*A*) Mean liquid saturation of the sample over time. (*B*) Gas connectivity: χ, gas phase Euler characteristic normalized by the dry pore space Euler characteristic and Γ, a measure of the cluster connectivity. Percolation threshold is at χ≈0. (*C* and *D*) Pore body filling statistics, calculated from logistic function fits to the mean gas composition in time for each pore body. (*C*) Arrival time. (*D*) Filling duration. For (*C* and *D*), the bar width equals 1 and 2 imaging time steps ($\Delta t^{\prime }=3.8$), respectively. The zoomed in box in (*D*) highlights ≈40 slow filling pores in experiment DG, representing ≈3% of all gas-filled pore bodies.

Quantitative analysis on the 3D gas–liquid arrangement in the rock is performed through the liquid saturation $\bar{S_{l}}$, and the gas connectivity, characterized by the Euler characteristic χ Eq. **2** and a percolation metric Γ Eq. **3** (*Materials and Methods*). This analysis confirms that experiment CG has a nearly constant saturation and gas phase connectivity, whereas PT and DG both exhibit decreasing liquid saturations and increasing gas connectivity over time throughout the experiment (Fig. 3 *A* and *B*). Fig. 3*B* highlights that nearly all of the gas phase in the pores is initially present as one connected cluster in experiment CG. This is expressed by the ratio of the main percolating cluster’s volume to the total gas volume: the “cluster connectivity” Γ≈1. In the other experiments (PT and DG), a dominant percolating gas cluster only develops over the course of the nitrogen injection, as water is displaced from the rock. For PT, this happens rapidly, as its initial saturation is close to the percolation threshold. Experiment DG, starting off from a lower gas saturation, has the most pronounced connectivity evolution. The proposed picture of the evolution of the gas–liquid distribution in the experiments is confirmed by the normalized Euler connectivity χ in Fig. 3*B*. This measure captures the internal connectivity of the gas clusters, with negative values representing a highly fragmented gas distribution and a value of 1 signifying that the gas is equally well connected as the full pore space of the rock.

To investigate how representative these fluid redistribution and gas mixing observations are for the entire sample, we analyze the pore scale displacement statistics. To this end, we calculate for each individual pore at which time the injected gas arrives (the “arrival time”) and how long the gas composition remains transient before reaching a steady-state in the pore (the “filling duration”). The detailed calculation is described in *Materials and Methods*. For experiment CG, the arrival times and filling durations are tightly distributed (Fig. 3 *C* and *D*), with the small range determined by the spatial distribution of pores along the vertical axis. This means that the arrival of the injected gas takes place in order of distance from the inlet, and that from the moment of this arrival, the gas composition changes smoothly and at nearly equal rate in all pores. This behavior is similar to the liquid solute transport observed in experiments described in the literature (20). This similarity can be traced back to the near-constant liquid saturation and gas connectivity during this experiment, highlighted in Fig. 3 *A* and *B*, respectively.

Conversely, experiments PT and DG, with coevolving liquid distribution and gas composition, show markedly different behavior, with bimodal arrival times and a large range of filling durations. Bimodal arrival times are caused by the connectivity evolution of the gas phase. The first peak ($t^{\prime }\approx 10$ to 15 in Fig. 3*C*) is associated with initial immiscible displacement events of pure nitrogen into water, and early reconnection of the gas phase, while it is still poorly connected (χ≈0). The second peaks ($t^{\prime }\approx 30$ in experiment PT and $t^{\prime }\approx 60$ in experiment DG in Fig. 3*C*) coincide with rapid changes in connectivity as the two gas components connect at the percolation threshold; χ increases from ≈0 to ≈0.2, and Γ rapidly approaches 1, indicating individual gas clusters connecting to a main percolating pathway. Contrary to experiment CG, the arrival times in experiments PT and DG are not ordered in function of distance to the inlet. This highlights the role of capillary fingering process in gas-water displacement (21): at this scale, gas displaces water from pores in a ramified pattern across the entire experimental field of view, rather than in a compact 1D front that moves longitudinally through the sample.

Similar to the arrival times, the filling durations of experiment PT and DG show several distinct peaks which represent characteristic events driven by the capillary-dominated immiscible fluid displacement. First, there exist many rapid filling events with characteristic filling durations of $t^{\prime }<15$, i.e. <4 imaging time steps. Experiment DG has a significantly higher frequency of events in this range than experiment PT. Given the filling duration definition and partial volume image artifacts, these events are effectively at, or under, the experimental temporal resolution. Analyzing the time-lapse images reveals these events are typically cascade (or avalanche) events occurring over multiple pores, interspersed by periods of slower movements, typical of Haines jumps (10, 22). This form of displacement is often characterized by “intermittent” dynamics and bursts in a slow drainage regime (23). Haines jumps can account for more than half of the volume displacement (10), cascading through multiple pore bodies with local velocities multiple orders of magnitude greater than the bulk flow rate (17). These rapid rearrangements bring the two different gases into contact with each other on a much shorter time-scale than bulk advection–diffusion would suggest, and with much larger composition gradients. Indeed, for the system here, the time-scale of a Haines jump across a pore of characteristic radius r is $\Delta t\propto {\left(\rho r^{3}/\gamma \right)}^{1/2}=0.1$ ms (10), which is significantly faster than both the characteristic advective time-scale, Δt∝r/u=10 s and diffusive time-scale, $\Delta t\propto r^{2}/D=40$ ms.

Alongside the rapid filling events, there exist many slower filling duration events with peaks around $t^{\prime }\approx 90$ to 100 for experiment PT and DG, and again at $t^{\prime }\approx 250$ for experiment PT. In fact, the distribution is more uniform for experiment PT compared to DG. Experiment PT has a saturation and connectivity which continually increases in time, as opposed to experiment DG which plateaus around $t^{\prime }\approx 100$ (Fig. 3 *A* and *B*). This continually evolving connectivity increases the sampling of the pore space, and the heterogeneous flow field induced by liquid saturations <1, leading to greater variance in the filling durations.

### 1.2. Bypassing of Trapped Gas Inhibits Mixing and Induces Ripening.

Further analysis of the individual pores reveals the creation of trapped krypton clusters, which are bypassed by the main nitrogen percolation; an example is shown in Fig. 2. Bypassing of trapped gas is most prominent in experiment DG, in which several smaller clusters remain disconnected for the entire experimental duration. When coupled with a spatially heterogeneous distribution of trapped krypton gas, as in experiment DG, the newly injected nitrogen gas can bypass certain regions, leaving regions of trapped krypton unconnected from the main gas body, separated from it by water.

Although the defending, trapped gas clusters are isolated from the main gas cluster, their composition is nevertheless observed to slowly change in time. Examples are shown in Fig. 2*B* for a specific pore filling event, and by the zoomed box in Fig. 3*D* for several slow filling events in experiment DG. A similar behavior is observed for all trapped clusters, which make up approximately 3% of the gas invaded pore bodies in experiment DG. This composition change is likely induced by dissolution and exsolution of the gas components in the water, in a form of multicomponent gas ripening (i.e. Ostwald ripening). Prior to the experiment, the water is saturated with nitrogen at experimental conditions to avoid gas dissolution in the flow lines before entering the sample. When the large-surface area of the trapped krypton clusters are exposed to this water, the krypton favorably dissolves into the water phase and the nitrogen exsolves into the gaseous phase. This follows Henry’s law for each gas component i: $c_{aq,i}=H_{i}p_{g}$, where $c_{aq,i}$ is the aqueous phase concentration, $H_{i}$ is Henry’s law constant and $p_{\mathrm{gi}}$ is the gas component partial pressure.

The exsolution of nitrogen occurs rapidly, since $p_{g_{N_{2}}}=0$ in the trapped gas ganglia initially. The dissolution of krypton occurs favorably over nitrogen, due to the higher value of $H_{\mathrm{Kr}}=2.5\cdot 10^{3}$ mol/kg · bar compared to $H_{N_{2}}=0.6\cdot 10^{3}$ mol/kg·bar at 298 K, 1 bar, and the ratio of molecular mass ($M_{\mathrm{Kr}}/M_{N_{2}}\approx 3$), meaning the amount of dissolved krypton at equilibrium would be ≈12× that of nitrogen. In our experiments, the trapped clusters slowly decrease in size as krypton is dissolved. An equilibrium cluster size and concentration would theoretically be reached when the partial pressure of nitrogen and krypton in the disconnected clusters equals the concentration in the water phase, assuming there is enough free-phase krypton to saturate the water phase.

### 1.3. The Pore Scale Coevolution of Saturation, Connectivity, and Composition Govern Continuum Mixing Dynamics.

**Fig. 4.**
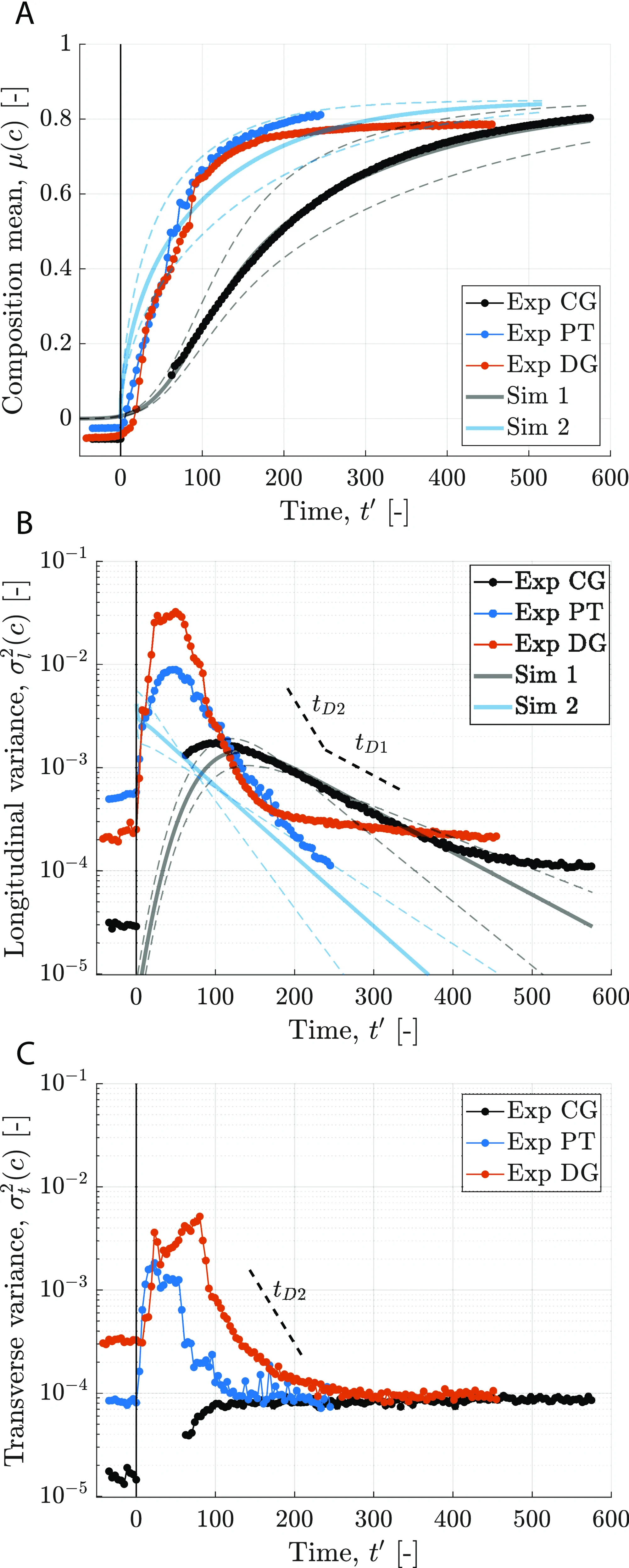
Continuum-scale mixing dynamics. (*A*) Sample mean gas composition in time. (*B*) Longitudinal variance in time. (*C*) Transverse variance in time. Thin dashed black & blue lines show results with saturation varied by ±0.2. The thick dashed black lines indicate confined diffusion scaling following $\sigma^{2}∼e^{-2t/t_{D}}$. The characteristic diffusion times, $t_{D1}$ and $t_{D2}$, are calculated using length scales of L= 7.5 mm and L= 3 mm, respectively, and saturations (impacting tortuosity) of $S_{g}=0.8$ and $S_{g}=0.5$, respectively. Note, the simulation transverse variance is equal to zero at all times. See Eq. **6** for variance definitions.

To quantify the temporal dynamics of gas mixing, we investigate the sample-average gas composition and variance as measures for the continuum (i.e. Darcy scale) mixing—see Fig. 4. We analyze both the longitudinal and transverse composition variances (Eq. **6**), which quantify fluctuations parallel and perpendicular to the mean flow direction, respectively.

As a reference model, reflecting the standard modeling approach, we compare these experimental results to the solution of a macroscopic 1D advection–diffusion equation (ADE) with multizone formulation (to capture the tubing and sample). This model provides a baseline to understand how the experiments deviate from the classical mixing behavior with the assumption of an immobile gas–liquid distribution under smooth diffusive mixing. It accounts for partial saturation effects by incorporating changes to the effective velocity and diffusivity, the latter through modifying the tortuosity. Although this approach assumes a constant saturation in time, solutions of the model at varying saturation levels enable us to compare our experimental results to the classical modeling framework. The full model derivation is described in *SI Appendix*.

The reference ADE model is used to describe the temporal evolution of the mean gas composition in experiment CG (Sim 1 in Fig. 4*A*), showing a good agreement with experimental data (see *Materials and Methods* for the model parameters). Initially the tubing is filled with krypton, c=0, meaning there is significant diffusive mixing of nitrogen and krypton en-route to the sample inlet. The sample boundary condition therefore exhibits a slowly increasing nitrogen composition in time. We then use the same parameters to describe the evolution of the mean gas composition in experiments PT and DG. These experiments show a sharp increase in gas composition (Fig. 4*A*), as near pure nitrogen hits the sample inlet directly behind the previously injected water. Hence, there is no nitrogen–krypton mixing in the tubing. To account for this boundary condition, the multizone model is adapted by initializing the tubing composition to be c≈1, i.e. nitrogen filled. The resulting model captures the general experimental behavior with no additional fitting (Sim 2 in Fig. 4*A*), but lacks the discrete, intermittent jumps in composition that are characteristic of experiment PT and DG.

The ADE model provides a good prediction of the evolution of the longitudinal variance for experiment CG (Sim 1 in Fig. 4*B*). The maximum variance occurs after the characteristic advection time through the saturated sample—due to significant diffusion and an increasing inlet composition—at $t^{\prime }\approx 100$. After this point, the variance decays exponentially, which can be rationalized as follows. After breakthrough, the gas composition close to the outlet increases (*SI Appendix*, Fig. S7), leading to a decay of the average longitudinal gradient by diffusion. This acts as diffusion in a confined domain (24), which reduces composition differences with a characteristic diffusion time scale $t_{D}=L^{2}/D_{\mathrm{eff}}$, with L a longitudinal length scale (found here to be L≈ 7.5 mm, just larger than the image region of $L_{i}=5.8$ mm) and $D_{\mathrm{eff}}=\phi S_{g}\tau D_{b}$ (25). Here, $S_{g}=0.8$ is the gas saturation, τ is the tortuosity factor approximated by a Bruggeman type formulation, $\tau =\phi^{1/2}S_{g}^{1/2}$ and $D_{b}$ is the bulk diffusivity. Since the variance evolves with the square of composition difference, it is expected to decay exponentially as $\sigma^{2}∼e^{-2t/t_{D}}$. This exponential decay is in good agreement with the experimental data (see the $t_{D1}$ black dashed line in Fig. 4*B*).

In complement to the longitudinal variance, we measured the transverse variance (Fig. 4*C*), which informs on variability of composition at a given longitudinal position. As these fluctuations are inherently not described by the reference ADE model, their occurrence is a direct assessment of the limits of the classical modeling approach. Experiment CG has effectively constant transverse variance, reflecting the experimental noise level. This confirms the one-dimensional nature of the flow in this experiment that is well described by the reference ADE model.

The mixing dynamics observed in experiments PT and DG are clearly distinct from experiment CG, and not well described by the classical ADE model, even when accounting for saturation effects (Sim 2 in Fig. 4*B*). The initial longitudinal variance buildup and decay ($t^{\prime }<150$) is much more rapid in these experiments compared to the reference ADE model, and experiment DG shows a slow decay in variance at late times ($t^{\prime }>150$). Both experiment PT and DG also exhibit large changes in transverse variance which are not predicted by the reference ADE model.

**Fig. 5.**
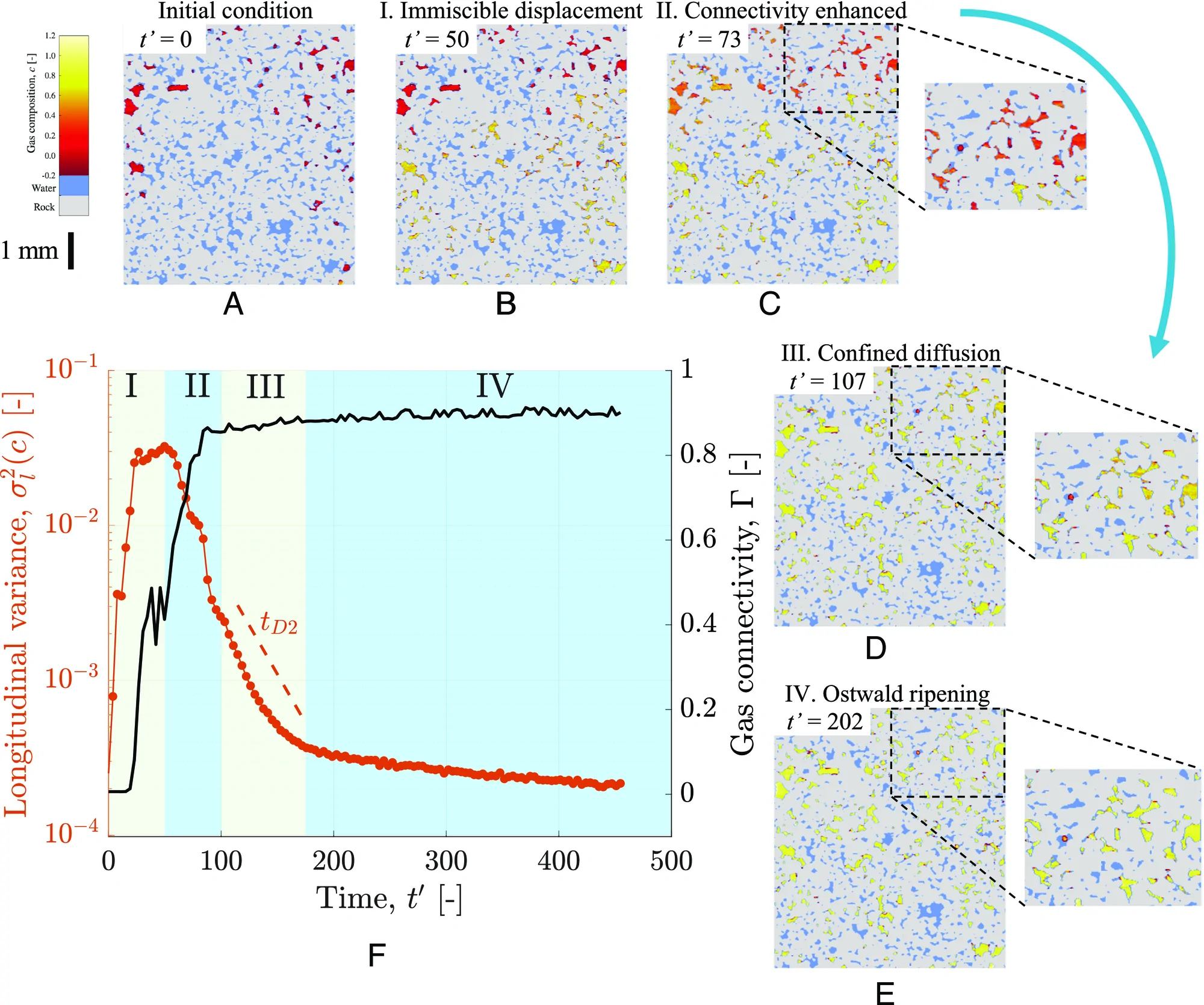
Mixing regime evolution for experiment DG. (*A*–*E*) 2D pore scale gas composition evolution at times representative of each mixing regime, along with the initial condition. The 2D cross-section orientations are the same as those in Fig. 1 with the inlet cropped. (*F*) Longitudinal composition variance, $\sigma_{l}^{2}\left(c\right)$, and gas cluster connectivity, Γ, for experiment DG with the mixing regime periods highlighted.

Our data reveals that when saturation & connectivity coevolve with mixing, the latter evolves through four distinct regimes. We define these regimes as I) immiscible displacement, II) connectivity enhanced, III) confined diffusion, and IV) Ostwald ripening (Fig. 5). We first describe these regimes for experiment DG below, comparing to experiment PT after, which follows a similar, but less pronounced mixing evolution.

I. Immiscible displacement: At early times, nitrogen displaces immiscibly into the resident brine, with some limited mixing with the residual krypton (transition from Fig. 5*A*–*B*). Hence, nitrogen and krypton phases coexist in the domain while remaining disconnected due to the presence of the liquid phase (Fig. 5*B*). This heterogeneous gas composition landscape leads to a build up of longitudinal composition variance (Fig. 5*F*, regime I), which peaks when the invading nitrogen breaks through. This occurs approximately at the percolation threshold $t^{\prime }\approx 25$ (when χ becomes greater than 0 with Γ≈0.4 in Fig. 5*F*).
II. Connectivity enhanced: Above the percolation threshold (χ>0, Γ>0.4), the two gas phases connect with each other, which creates intermediate composition levels (see orange clusters in Fig. 5*C*). This leads to a regime of rapid mixing as variance decays by an order of magnitude between $t^{\prime }\approx 50$ to 100, with several discrete jumps in variance visible in Fig. 5*F*. During this period we also observe a sharp increase in the connectivity (Γ in Fig. 5*F*). The connectivity enhances mixing by rapidly bringing into contact pores with contrasted gas composition, which then mix quickly by diffusion (see Fig. 2*A* Pore 1, Exp DG, and transition from Fig. 5*C*–*D*). The discrete jumps in variance are indicative of intermittent dynamics and bursts in a slow drainage regime (23), which can mix the gas components rapidly, faster than bulk advection or diffusion would suggest (Section 1.1).
III. Confined diffusion: After $t^{\prime }\approx 100$, the connectivity reaches a plateau (Γ→0.9 in Fig. 5*F*). In this regime, we observe a much smoother, gradual decay in variance, which follows an approximate exponential decay. We interpret this evolution as a signature of confined diffusion, where diffusion slowly homogenizes composition levels through the fully connected network of gas clusters (transition from Fig. 5 *D* and *E*). The subtle variations in the transverse composition compared to longitudinal composition is visible in the zoomed *Insets* in Fig. 5 *D* and *E*. The characteristic time of the exponential decay $t_{D2}=L^{2}/D_{\mathrm{eff}}$ is consistent with a characteristic diffusion length equal to the transverse sample radius L=3 mm, as opposed to the longitudinal length which governs experiment CG. Furthermore, we used a saturation of $S_{g}=0.5$ (indicative of experiment DG) to calculate the tortuosity factor and $D_{\mathrm{eff}}$ (as opposed to $S_{g}=0.8$ for experiment CG) which reduces $D_{\mathrm{eff}}$ by a factor of 2.
IV. Ostwald ripening: At late times ($t^{\prime }\geq 175$), we observe a slow decay in variance (Fig. 5, regime IV). We interpret this shutdown in mixing rate as a result of bypassed, trapped gas (e.g. the trapped ganglia to the mid-left of zoomed *Insets* in Fig. 5*C*–*E*). The slowly declining variance indicates that the discrete, trapped gas clusters are still mixing, indicative of the multicomponent Ostwald ripening process observed in the pore scale gas composition field. This is visible in the zoomed *Insets* in Fig. 5 *D* and *E*.

Mixing regimes I, II, and III are also observed in experiment PT (Fig. 4*B*), albeit in a reduced manner compared to experiment DG, due to the differences in multiphase flow. Experiment PT is initialized near the percolation threshold (χ is just above zero in Fig. 3), meaning there are fewer immiscible displacement events before the gas connects across the image region compared to experiment DG. This means the maximum variance obtainable in experiment PT is reduced compared to DG, and the approach to the maximum is smoother due to simultaneous advective and diffusive mixing across the gas network. The rapid decay of variance during the connectivity enhanced mixing period (regime II in Fig. 5) is less sharp in experiment PT compared to DG. This is due to the more gradual increase in cluster connectivity observed in Experiment PT (see Γ in Fig. 3*B*), with fewer intermittent bursts or large redistribution events. Once Γ→1 at t≈100 in experiment PT, we observe a similar gradual decay in variance as experiment DG in regime III. This decay is characterized by the same exponential behavior—a confined diffusion process—in both experiments, due to their similar saturation states, tortuosity, and diffusive length scales governed by the initial percolation process. Finally, we observe a continued gradual decline in variance in experiment PT at late time, in contrast to the plateau in experiment DG, which defines regime IV. There is little bypassed gas in experiment PT due to the initial gas distribution and subsequent multiphase flow, meaning once the gas is well connected, mixing proceeds in a confined diffusion process until the end of the experiment.

## 2. Discussion

Our experiments explore element transport in the evolving gas-phase, as opposed to the liquid phase most commonly investigated (11, 13–15). This feature was chosen to analyze the case of gas mixing in unsaturated porous media, with specific relevance to UHS and CCS in depleted gas fields. Our results reveal the impacts of multiphase flow on gas mixing in porous media during the coevolution of the composition (i.e. concentration), saturation and connectivity. Coevolution is the prevailing regime in many multiphase flow systems, as noted by ref. 16, and indeed prevails for most of the injection period in field-scale storage operations (26).

Recent studies (16, 27) have shown numerically that dynamic connectivity during multiphase flows can accelerate spreading and mixing of solutes compared to single-phase flow. This is due to repeated activation and deactivation of flow pathways, even if the flow is globally at steady state (28, 29). Our unsteady-state experiments reveal a different mechanism by which mixing is controlled by the interplay between compartmentalization and intermittent connectivity changes. These multiphase filling events (e.g. Haines jumps) connect clusters with contrasted gas compositions that then mixing rapidly by diffusion. In these situations, current models break down when attempting to capture the connectivity driven mixing dynamics, even when accounting for partial saturation effects. Inherently, these continuum models cannot capture both the longitudinal and transverse variance decay associated with connectivity-driven mixing events occurring intermittently across the field of view.

We observe a shutdown in these rapid mixing dynamics at late-times, associated with trapped gas clusters. A slow change in the composition of the gas clusters occurs through gaseous exchange with the liquid phase, in a form of Ostwald ripening. Theoretically, multicomponent gas ripening occurs in two evolution phases (30); a partitioning phase, whereby mass transfer is driven by composition variation, and a coevolution phase whereby mass transfer is driven by capillary pressure equilibration in the bubbles. In our experiments we observe the partitioning phase of this ripening process, whereby compositional nonequilibrium between the gas and brine phase leads to the exsolution of nitrogen, and dissolution of krypton into the brine phase. The dissolved krypton is transported away by fluid flow, implying that equilibrium is not established and the trapped ganglia slowly shrink in time.

### 2.1. Macroscopic Upscaling.

Our analysis highlights the range of validity, and breakdown, of classical continuum models in describing gas mixing evolution in multicomponent multiphase flow in porous media. These findings open perspectives for upscaling the effect of pore scale multiphase flow on gas mixing, with dominant mechanisms that differ in the four mixing regimes identified in Fig. 5.

First, for periods of evolving gas connectivity below the percolation threshold (regime I in Fig. 5), the increase in composition variance is driven mostly by pore scale unsteady multiphase flow with little mixing of the gases. The peak variance depends on the initial distribution of the resident gas, and the displacement regime (capillary fingering, compact displacement, viscous fingering, etc.). One would therefore need to capture the pore scale fingering dynamics to capture the macroscopic composition variance evolution. Upscaling capillary and viscous fingering regimes is the focus of on-going research, but recent results have indicated promising avenues with extensions to Darcy’s law (31, 32).

Above the percolation threshold, the gas cluster connectivity and saturation continue to coevolve. We find a large part of gas mixing occurs during this period with rapid connectivity change (regime II in Fig. 5). We hypothesize that the additional physics needed to capture these mixing dynamics are largely associated with multiphase flow dynamics—intermittency (26) and ganglion dynamics (33)—that deviate from connected pathway flow as described by classical multiphase Darcy’s law. Quasi-static approaches may incorporate concepts from percolation theory to link the cluster size and connectivity evolution with saturation (34). For a fully dynamic model, interfacial information may need to be incorporated at the continuum scale through e.g. a thermodynamic approach (35, 36) or through geometric state approaches utilizing topological information directly (37).

When the gas-phase cluster connectivity is near constant (regime III in Fig. 5), mixing is well characterized by a confined diffusion process at late time. Scaling the effective diffusivity through saturation and porosity (i.e. tortuosity), and using a transverse as opposed to longitudinal length scale reproduces the observed late-time diffusive scaling behavior between experiment PT/DG and CG. This highlights that when the gas phase is connected with a continuous percolating network at the REV scale (i.e. the cluster connectivity Γ→1), classical diffusion plays out at late time, even with changing saturation and connectivity at the pore–scale (i.e. χ can still be changing as is seen in experiment PT for the whole experiment). We find that the diffusive mixing length scale is governed by the initial immiscible displacement process; in experiments PT/DG the longitudinal network readily connects at percolation, after which connections in the transverse direction and subsequent diffusion control the mixing, whereas in experiment CG the gas is always fully connected across both the transverse and longitudinal directions, so diffusive mixing is limited by the larger length scale, i.e. longitudinal. This implies that the macroscopic diffusive length scale is primarily governed by the percolating cluster length scales. To upscale this behavior, a classical ADE model would have to be scaled by tortuosity and the percolating cluster lengths to capture the macroscopic transport in Regime III.

Finally, to upscale the multicomponent gas ripening behavior, as observed in the late stages of experiment DG (regime IV), it may be possible to extend the work of Li et al. (38) to a multicomponent system, using a retardation factor to modify the effective aqueous diffusion rate. This would allow a relatively simple extension to multiphase Darcy’s law with a suitable equation of state. For a more complete thermodynamic description, kinetic theory could be utilized to upscale the impacts into a continuum model, as described recently (39).

The above approaches may present viable avenues for capturing the macroscopic mixing process across the four regimes, but their validation with experimental data, and unification into a single model is the focus of on-going research. In the present work, we note that the time-scale of the deviations from the classical mixing behavior is relatively short, largely due to experimental limitations in the macroscopic spatial scales that are observable while still resolving the pore–scale gas composition field. Future work would look to extend these scales, and perform controlled experiments on the system for the end-member states, i.e. a system setup purely for multicomponent ripening without background flow, and a system setup solely for connectivity mediated mixing. Coupling the experimental methodology here with recently developed particle image velocimetry (17) could also provide more information on local velocities during the connectivity driven mixing.

### 2.2. Implications for Underground Hydrogen Storage.

Our observations are a step in understanding unsteady multicomponent transport during multiphase flow in porous media. The experiments were specifically designed to mimic the conditions encountered in underground hydrogen storage, where gas mixing results in reduced hydrogen purity upon gas production. Previous work has identified that the predominant mechanism for gas mixing during UHS stems from large-scale heterogeneity in the reservoir, i.e. permeability variance and associated macroscopic dispersion (8, 40, 41). During the storage phase, molecular diffusion also acts to mix the gases (42). Our results indicate that the pore–scale connectivity of the gas phase may also play an important role, acting to control mixing at the leading edge of the plume and in regions where connectivity is evolving in time. This effect would be amplified by macroscopic heterogeneity, due to variations in saturation and connectivity through capillary heterogeneity (43). In our experiments, the connectivity evolution is relatively brief due to the length-scale of the setup, but during UHS this time-period could be a significant portion of the field operation. The multicomponent gas ripening effects observed in our experiments also have consequences at the field scale. Any hydrogen that was trapped, or left remaining in a free-phase after the production period, would start to chemically equilibrate with the resident brine. The latter would be saturated with natural gas due to prolonged (geological time-scale) exposure. The hydrogen gas phase would therefore be reduced in purity, and impact subsequent injection/production cycles. The time-scale of this equilibration process could be relatively fast at the field scale due to the compositional imbalance of the free-phase gas and resident brine.

## 3. Materials and Methods

We use a cylindrical Bentheimer sandstone sample with diameter, Φ=6 mm, length, $L_{s}=26.2$ mm, imaged porosity ϕ=0.18 and permeability $k_{\mathrm{abs}}=565$ mD. The sample is mounted vertically in a Viton rubber sleeve, within a Hassler-type X-Ray transparent PEEK core-holder. The confining pressure on the sleeve is maintained at 4.6 MPa throughout, to eliminate fluid bypass around the sample. The core-holder is connected to inflow/outflow pumps through several PEEK tubing lines and valves, allowing the injection/withdrawal of nitrogen, krypton, and water fluid phases at controlled rates and pressure. The pore-pressure is maintained at ($\bar{P}=3.57$ MPa, σ(P)=0.029 MPa). The temperature was ($\bar{T}=22.6$ °C, σ(T)=0.17 °C). The full experimental setup is detailed in *SI Appendix*.

In all three flow experiments, the sample is initially saturated with water doped with potassium iodide (20% wt. KI in deionized water). Following this, three different initialization procedures are used to set up the krypton saturation and connectivity to mimic the field conditions described in the text, see Table 1. Nitrogen is then injected at a constant flow rate (Q=0.003 ml/min) with simultaneous X-Ray micro-CT imaging to track the gas mixing and multiphase flow inside the core.

X-Ray micro-CT imaging was performed with the Environmental Micro-CT (EMCT) scanner in the Centre for X-Ray Tomography (UGCT) at the University of Ghent. The EMCT scanner featured a rotating cone-beam source and detector, with a fixed sample, allowing fast, stable X-Ray CT scanning. We used a scanning energy of 130 kV, a power of 16 W, a 20 ms exposure and 1,001 projections for each tomographic scan. We used 2× averages for each projection, resulting in a 40 s acquisition time per 3D time step image. This was repeated 70 to 150×, obtaining a time series of 3D images for each experiment. The spatial resolution was 12.6 μm with a field-of-view of 8 × 8 × 8 mm.

Each batch of 1,001 projections were reconstructed using the Panthera software from Tescan, with automated center of rotation retrieval. After reconstruction, the images are registered in Avizo using normalized mutual information, and then normalized from 16 bit to 8 bit using $CT_{8bit}=S\left(CT_{16bit}+O\right)$, with S=0.006375 and O=−15,000. Images were then processed using the ImageProcessing toolbox in Matlab; nonlocal means filtering (search window size 15, comparison window size 5, smoothing level 15), threshold segmentation (grayscale values <120 out of 255 assigned gas phase) and small-cluster removal using the connected components bwconncomp function (clusters <9 voxels removed). Gas saturations were calculated directly from the segmented image volume fractions.

To calculate gas compositions, calibration experiments were initially performed. The sample was saturated with krypton or air (/nitrogen) and the partial pressure varied incrementally up to the working pressure, with images acquired intermediately. A linear relationship was found between the partial pressure and attenuation (see the SI for details). Assuming ideal gas behavior (both gases exhibit <8% variation in real gas density versus ideal gas density over the experimental conditions) and near-constant experimental pressure and temperature, the voxel-wise gas composition, c, in the pore space can therefore be calculated from the calibration images using a linear mapping:

[1] $$\begin{array}{c} c=\frac{I_{kr=1}-I_{\mathrm{experiment}}}{I_{kr=1}-I_{n2=1}}, \end{array}$$

where $I_{kr=1}$, $I_{n2=1}$, and $I_{\mathrm{experiment}}$ are the reconstructed grayscale intensities for the krypton calibration image, nitrogen (or air) calibration image and the experimental image, respectively.

### 3.1. Pore Scale Metrics.

The Euler characteristic, χ, is a topological invariant which measures the connectivity of the pore space. We calculate it using the imEuler3D function from the imMinkowski package with a 3D connectivity of 26 (44, 45):

[2] $$\begin{array}{c} \chi =V-E+F, \end{array}$$

where V are the number of vertices, E are the number of edges and F are the number of faces in the segmented binary image. We normalize the gas phase $\chi_{g}$ by the whole pore space $\chi_{p}$, i.e. $\chi =\chi_{g}/\chi_{p}$. χ<0 indicates a disconnected gas phase, which percolates as χ≈0, and becomes strongly connected as χ→1.

We also calculate Γ, a percolation metric detailed in refs. 46 and 47:

[3] $$\begin{array}{c} \Gamma =\frac{1}{n_{g}^{2}}\sum_{i=1}^{N}n_{i}^{2}, \end{array}$$

where, $n_{g}$ is the total number of gas phase voxels, N is the total number of distinct gas clusters, and $n_{i}$ is the number of voxels in each gas cluster, i. As Γ→1, the gas phase is connected in increasingly fewer clusters, approaching a single cluster spanning the pore space.

To calculate pore-filling statistics, the pore space is first divided into pore bodies using the algorithm of ref. 48, generating 2901 bodies and 8,276 connecting pore throats. The image based voxelized gas compositions are mapped onto each pore body to generate averaged pore body gas compositions in time. From this, a 4 parameter logistic function is fitted to the data:

[4] $$\begin{array}{c} c_{b}=x\left(4\right)+\frac{x(1)-x(4)}{{\left(1+t/x\left(3\right)\right)}^{x(2)}}, \end{array}$$

where $c_{b}$ is the pore body average composition, t is time, and x(1),x(2),x(3), and x(4) are the fitting parameters representing the minimum gas composition, slope, inflection point, and maximum gas composition, respectively. The model is fitted to the gas composition-time data using the *fmincon* function in Matlab, which minimizes the RMSE between model and data using an interior-point algorithm. The minimum composition is constrained at x(1)=0, the maximum is bounded by x(4)=1, and is generally higher than the final experimental gas composition since it represents the infinite time solution.

We determine the characteristic arrival time for each pore body as the first time point when $c_{b}>0.1\cdot x\left(4\right)$. Similarly, we calculate each pore body’s transient filling duration as the time for $c_{b}$ to increase from 0.1·x(4) to 0.9·x(4). Note, for experiment CG, the earliest arrival times are $t^{\prime }\approx$ 30 not $t^{\prime }\approx$ 0 due to the arrival time definition of $c_{b}>0.1\cdot x\left(4\right)$ and the highly diffusive nature of the flow.

### 3.2. Nondimensional Scaling.

Time, t, is nondimensionalized with a characteristic pore scale time, $t_{c}$ using the characteristic pore length, $l_{c}$, and interstitial pore scale velocity, u:

[5] $$\begin{array}{c} t^{\prime }=\frac{t}{t_{c}}=\frac{\mathrm{tu}}{l_{c}} \end{array}$$

For our experiments—with $l_{c}=104$ μm from pore network extraction, and $u=Q/\left(A\phi \right)=9.93\times 10^{-6}$ m/s where A is the sample cross-sectional area, Q is the volumetric flow rate of nitrogen (Q=0.003 ml/min) and ϕ=0.178 is the imaged porosity—$t_{c}=l_{c}/u=10.5$ s, meaning each scan captures the average movement over ≈ 4 characteristic pore lengths. The experimental time is calculated from the point at which the mean gas composition variance first increases past the baseline variance (i.e. 100% krypton composition for each experimental state), signifying nitrogen has entered the sample. For experiment CG, several scans were missed at the start of the nitrogen injection due to scanner failure, so the start time is back-calculated using the simulation results and baseline variance before injection, with a maximum uncertainty estimated at ≈5 min (±3% of the total experimental time). Note, for exp CG, we shift the final 10 baseline scans before the scanner failure up to t=0 in the figures to indicate the initial conditions.

The pore scale capillary number is given by $Ca=u\mu_{g}/\gamma =2.6\times 10^{-9}$, where $\mu_{g}=1.83\times 10^{-5}$ Pa.s is the nitrogen viscosity (49), and γ=70.1 mN/m is the nitrogen-water interfacial tension (50), with minor variance for krypton-water or brine fluid pairs. The macroscopic capillary number is given by $N_{c}=H\Delta P/L_{s}\Delta P_{c}$ = 0.23 (51), where H = 6 mm is the sample diameter, $L_{s}=26.2$ mm is the sample length, ΔP≈5 kPa is the viscous pressure drop over the sample length, and $\Delta P_{c}=5$ kPa is the characteristic capillary pressure variation (52). The pore scale Péclet number is given by $Pe_{l}=ul_{c}/D_{b}=3.4\times 10^{-3}$ where $D_{b}=3.0\times 10^{-7}$ m^2^/s is the bulk diffusion coefficient between nitrogen and krypton. The continuum-scale Péclet number is $Pe_{g}=u\phi L_{s}/D_{\mathrm{eff}}=2.87$ where $D_{\mathrm{eff}}$ is the effective continuum-scale diffusion coefficient between nitrogen and krypton, see *ADE* below. The Reynolds number is given by $Re=\rho_{g}ul_{c}/\mu_{g}=2.3\times 10^{-3}$, where $\rho_{g}=41.2$ is the nitrogen density (49).

### 3.3. Variance Calculations.

The longitudinal and transverse variances are calculated respectively as

[6] $$\begin{array}{c} \sigma_{l}^{2}=\frac{1}{N_{l}}\sum_{i=1}^{N_{l}}|{\left\langle c_{i}\right\rangle }_{l}{-\mu |}^{2},\;\;\;\;\;\;\sigma_{t}^{2}=\frac{1}{N_{t}}\sum_{i=1}^{N_{t}}{\left|{\left\langle c_{i}\right\rangle }_{t}-\mu \right|}^{2}, \end{array}$$

where $N_{l}$ and $N_{t}$ are the total number of measurement points in the longitudinal and transverse directions, respectively. ${\left\langle c_{i}\right\rangle }_{l}$ and ${\left\langle c_{i}\right\rangle }_{t}$ are the mean compositions at point i, averaged over the x-y coordinates at 100 μm increments along the z-axis (longitudinal) and the y-z coordinates at 100 μm increments along the x-axis (transverse), respectively. μ is the population mean:

[7] $$\begin{array}{c} \mu =\frac{1}{N}\sum_{i=1}^{N}c_{i} \end{array}$$

Note, the transverse variance is computed on the inscribed square of the circular core, so each ${\left\langle c_{i}\right\rangle }_{t}$ uses the same number of averaging points. The x-axis and y-axis transverse variances are very similar, so we report just the x-axis transverse variance.

### 3.4. 1D Modeling Based on the Advection Diffusion Equation (ADE).

We utilize a classical 1D advection–diffusion equation, with multizone formulation to capture the gas composition transport in the tubing and sample flow, given a constant porosity and saturation in each zone. Full details are given in *SI Appendix*. The formulation allows the advection & diffusion of nitrogen from the pressure vessel (constant gas composition boundary), through the tubing and into the sample. The simulation has four parameters with uncertainty: the tubing length, $L_{t}=42$ cm, sample porosity ϕ=0.24 (higher than image porosity to account for subresolution porosity), bulk diffusion coefficient of nitrogen-krypton, $D_{b}=3\times 10^{-7}$ m^2^/s [in-line with known noble gas diffusion coefficients at atmospheric conditions and ideal gas pressure scaling (53–55)] and the pressure vessel gas composition, $c_{v}=0.85$ (necessarily <1 due to back-diffusion of krypton from previous calibration experiments). We take the gas saturation as $S_{g}=0.8$, and vary it ±0.2 to account for variations between the experiments. The effective continuum diffusivity coefficient in the sample is given by $D_{\mathrm{eff}}=\phi \tau S_{g}D_{b}$ (25), with the tortuosity factor approximated by a Bruggeman type formulation, $\tau =\phi^{1/2}S_{g}^{1/2}$.

The unknown parameters are initially estimated from experimental measures and literature values, then refined through an ensemble sweep across the likely ranges, with the lowest $L_{2}$ error between simulation and experimental gas composition profiles determining the final values. The resulting parameters are nonunique, but can only vary within ≈20% to achieve similar convergence to the experimental results.

## Supplementary Material

Appendix 01 (PDF)

Movie S1. 2D rendering of the gas composition and gas-water distribution during experiment CG. We show an x-z cross-section taken through the centre of the injection port. Krypton gas (c = 0), nitrogen (c = 1), water (blue), rock (dark grey) and the PEEK inlet (light grey) are shown.

Movie S2. 2D rendering of the gas composition and gas-water distribution during experiment PT. We show an x-z cross-section taken through the centre of the injection port. Krypton gas (c = 0), nitrogen (c = 1), water (blue), rock (dark grey) and the PEEK inlet (light grey) are shown.

Movie S3. 2D rendering of the gas composition and gas-water distribution during experiment DG. We show an x-z cross-section taken through the centre of the injection port. Krypton gas (c = 0), nitrogen (c = 1), water (blue), rock (dark grey) and the PEEK inlet (light grey) are shown.

Movie S4. 3D rendering of the gas composition during experiment CG. We show a cutout of the full cylindrical sample taken through the centre of the injection port. Krypton gas (c = 0) and nitrogen (c = 1) are shown, the rock and water phases are made transparent.

Movie S5. 3D rendering of the gas composition during experiment PT. We show a cutout of the full cylindrical sample taken through the centre of the injection port. Krypton gas (c = 0) and nitrogen (c = 1) are shown, the rock and water phases are made transparent.

Movie S6. 3D rendering of the gas composition during experiment DG. We show a cutout of the full cylindrical sample taken through the centre of the injection port. Krypton gas (c = 0) and nitrogen (c = 1) are shown, the rock and water phases are made transparent.

Movie S7. 2D rendering of the gas composition and gas-water distribution during experiment CG. We show a zoomed in view around ‘Pore 1’, described in the main text. Krypton gas (c = 0), nitrogen (c = 1), water (blue) and rock (white) are shown.

Movie S8. 2D rendering of the gas composition and gas-water distribution during experiment PT. We show a zoomed in view around ‘Pore 1’, described in the main text. Krypton gas (c = 0), nitrogen (c = 1), water (blue) and rock (white) are shown.

Movie S9. 2D rendering of the gas composition and gas-water distribution during experiment DG. We show a zoomed in view around ‘Pore 1’, described in the main text. Krypton gas (c = 0), nitrogen (c = 1), water (blue) and rock (white) are shown.

Movie S10. 2D rendering of the gas composition and gas-water distribution during experiment CG. We show a zoomed in view around ‘Pore 2’, described in the main text. Krypton gas (c = 0), nitrogen (c = 1), water (blue) and rock (white) are shown.

Movie S11. 2D rendering of the gas composition and gas-water distribution during experiment PT. We show a zoomed in view around ‘Pore 2’, described in the main text. Krypton gas (c = 0), nitrogen (c = 1), water (blue) and rock (white) are shown.

Movie S12. 2D rendering of the gas composition and gas-water distribution during experiment DG. We show a zoomed in view around ‘Pore 2’, described in the main text. Krypton gas (c = 0), nitrogen (c = 1), water (blue) and rock (white) are shown.

## Data Availability

Data (raw + processed images, pressure, temperature, and flow rates) along with processing and simulation codes are openly available through Zenodo with a Creative Commons Attribution 4.0 International license (56).
