# Supplementary material for: Intermittent gas–liquid rearrangement controls the mixing of gases in porous media
Source: Proc Natl Acad Sci U S A. 2026 Nov 5;123(45):e2612721123. doi: 10.1073/pnas.2612721123 (PMC13752354; doi:10.1073/pnas.2612721123)
Supplement: Supplementary file 1 [file pnas.2612721123.sapp.pdf]

# Supporting information document for 'Intermittent gas-liquid rearrangement controls the mixing of gases in porous media'

S.J. Jackson[a,*], S. Ellman[b], G. Kalyoncu[b], T. Le Borgne[c,d,e], and T. Bultreys[b]

[a]Energy Research Unit, Commonwealth Scientific and Industrial Research Organisation (CSIRO), Clayton, Victoria, 3168, Australia
[b]Department of Geology and Centre for X-ray Tomography (UGCT), Ghent University, Ghent, Belgium
[c]Géosciences Rennes, Université de Rennes, CNRS, Unité Mixte de Recherche 6118, Rennes 35000, France
[d]The Njord Centre, Department of Geosciences, University of Oslo, Oslo 0316, Norway
[e]Institute of Environmental Assessment and Water Research (IDAEA), Spanish National Research Council (CSIC), Barcelona, 08034, Spain
[*]*Corresponding author email: samuel.jackson@csiro.au*

## S1 Experimental methodology

The experimental setup is shown diagrammatically in Fig. S1. A complex series of three-way and two-way valves allow the injection or withdrawal of nitrogen, krypton, brine or de-ionised (DI) water through the system. For accurate flow control of compressible gases at low rates, a Vindum pump (VP-Series dual-piston high-pressure pump) was used in a withdrawal mode at the outlet of the sample, as opposed to injecting at the inlet of the sample. When coupled with the pressure vessel at the inlet of the sample, the large volume in the pressure vessel (600 ml) meant that near constant pressure could be maintained (3.6 MPa) for the main nitrogen flow rates used in the experiments (0.003 ml/min). Krypton was used to setup an initial saturation in the sample, at higher rates (0.03 ml/min), meaning injection could be performed with adequate flow control using an ISCO pump 500D pump. A Teledyne Reaxus 6010R reciprocating pump was used for accurate confinement pressure control around the sleeve of the sample, operating at a constant 46 bar. We used an Equilibar Research Series back pressure regulator to control purging and cleaning of the sample.

The sample has a pore volume of $\approx$ 0.13 ml. The tubing connecting valve Vin2 to the sample inlet has a dead-volume of $\approx$ 0.25 ml, i.e. $\approx$ 2 pore volumes. This dead volume has to be displaced before the intended fluid enters the sample, meaning at the experimental injection rate of 0.003 ml/min, it takes $\approx$ 70-80 minutes from switching the valves until new fluid enters the sample. Although rates could be changed to traverse this dead volume more rapidly, pressure was stable at lower rates, meaning displacement times were more predictable, and scanning could be initiated just before fluid entered the sample. The full experimental protocols are listed below, starting with the initialisation procedure.

### Initialisation procedure

1. The core sample is placed in a Viton sleeve, and mounted in the PEEK core-holder.
2. A confining pressure of 4.6 MPa is applied with DI water to prevent fluid bypass around the sample.
3. The system is checked for leaks, ensuring no fluid has entered the sample and constant pressure can be maintained with near to zero flow.

4. Compressed air is purged through the sample using a separate air compressor, at 0.5 - 1 MPa for 24 hours to dry the sample.

5. A dry scan is performed, with the sample fully saturated with air.

6. Krypton gas is injected into the sample using the BP regulator to control pressure at 1 MPa. Many pore volumes are injected (>50), saturating the sample with krypton.

7. Calibration scans are taken for krypton at varying pore pressures, by raising the ISCO pump pressure incrementally in constant pressure mode. The pressure is allowed to stabilise in each case, so there is no flow in the sample. Scans are taken at corresponding pressures ranging across 1, 2, 3, 3.6 and 4 MPa.

8. The pressure vessel (600 ml) was filled with 20 wt.% KI brine and nitrogen in a 50/50 split.

9. Nitrogen was purged through the sample into the BPR, ensuring nitrogen saturated the tubing from the vessel top, valve Vv1, to valve Vin2.

10. The nitrogen bottle was then used to displace brine through the sample at high pressure flowing through the BPR at atmospheric conditions until no air bubbles were produced. This ensured brine saturated the tubing from the the bottom of the vessel, valve Vv2, to the sample inlet.

11. The valves were switched so that Vindum pump received fluid, as opposed to the BPR.

12. The transducer lines were purged with brine at low pressure.

13. The whole system was brought slowly up to experimental pressures (3.6 MPa) using the nitrogen bottle, and operating the Vindum pump in constant pressure mode. Pressure was incrementally increased to avoid over-pressuring the transducers and allowing aqequate time for nitrogen to flow into the pressure vessel. The pressure vessel was bought up to experimental pressure

14. The nitrogen bottle was closed. Brine was withdrawn through the system at constant rate by the Vindum pump connected to the pressure vessel, until stable pressure drops were recorded.

15. A brine saturated scan was acquired, ensuring no gas bubbles existed in the system. This is the initial condition for each of the three flow experiments.

Steps 1-15 were performed before the start of experiment CG. After experiment CG, the system pressure is reduced slowly to atmospheric conditions. Following this, steps 10 - 15 are performed to re-initialise the brine saturated samples for experiments PT and again for experiment DG. Brine is able to readily dissolve trapped in the sample, meaning the sample can be readily reverted back to a fully saturated system given enough pore volumes of injection. The experimental protocols for the experiments are listed below.

**Experiment CG flow procedure**

1. Krypton is injected into the sample. The Vindum pump is used in constant withdraw mode at high rate (0.03 ml/min) to 'pull' fluid through the outlet of the sample. The ISCO pump is operated at constant pressure mode, to supply krypton. Krypton is injected for $\approx$ 22 PV, achieving a gas saturation of $\approx$ 0.8.

2. The krypton flow rate is dropped to 0.003 ml/min for ≈ 2 PV, allowing the pressure to equilibrate across the sample.
3. Valves are switched so that the pressure vessel is connected to the inlet of the sample and nitrogen starts displacing into the tubing (from valve Vin2) and sample at constant rate 0.003 ml/min.
4. After 70 minutes of nitrogen flow, continuous scanning is started, in which 145 full rotation scans are performed.

**Experiment PT flow procedure**

1. Krypton is injected into the sample. The Vindum pump is used in constant withdraw mode at high rate (0.03 ml/min) to 'pull' fluid through the outlet of the sample. The ISCO pump is operated at constant pressure mode, to supply krypton.
2. The krypton flow rate is increase from 0.03 ml/min to 0.09 ml/min to 0.45 ml/min, and then injected for 3 PV to create a high saturation in the sample.
3. The krypton flow rate is decreased to 0.03 ml/min for 9 PV.
4. Valves are switched so that the pressure vessel is connected to the inlet of the sample and brine starts displacing into the tubing (from valve Vin2) and sample at constant rate 0.03 ml/min for 10 PV.
5. The brine flow rate is dropped to 0.003 ml/min for ≈ 1 PV, allowing the pressure to equilibrate across the sample.
6. Valves are switched so that the nitrogen starts displacing into the tubing (from valve Vin2) and sample at constant rate 0.003 ml/min.
7. After 70 minutes of nitrogen flow, continuous scanning is started, in which 75 full rotation scans are performed. Nitrogen hits the sample inlet ≈ 3 minutes after scanning has started.

**Experiment DG flow procedure**

1. Krypton is injected into the sample. The Vindum pump is used in constant withdraw mode at high rate (0.03 ml/min) to 'pull' fluid through the outlet of the sample. The ISCO pump is operated at constant pressure mode, to supply krypton. Krypton is injected for 9 PV.
2. Valves are switched so that the pressure vessel is connected to the inlet of the sample and brine starts displacing into the tubing (from valve Vin2) and sample at constant rate 0.03 ml/min for 20 PV.
3. The brine flow rate is dropped to 0.003 ml/min for ≈ 1.2 PV, allowing the pressure to equilibrate across the sample.
4. Valves are switched so that the nitrogen starts displacing into the tubing (from valve Vin2) and sample at constant rate 0.003 ml/min.
5. After 70 minutes hour of nitrogen flow, continuous scanning is started, in which 135 full rotation scans are performed. Nitrogen hits the sample inlet ≈ 2 minutes after scanning has started.

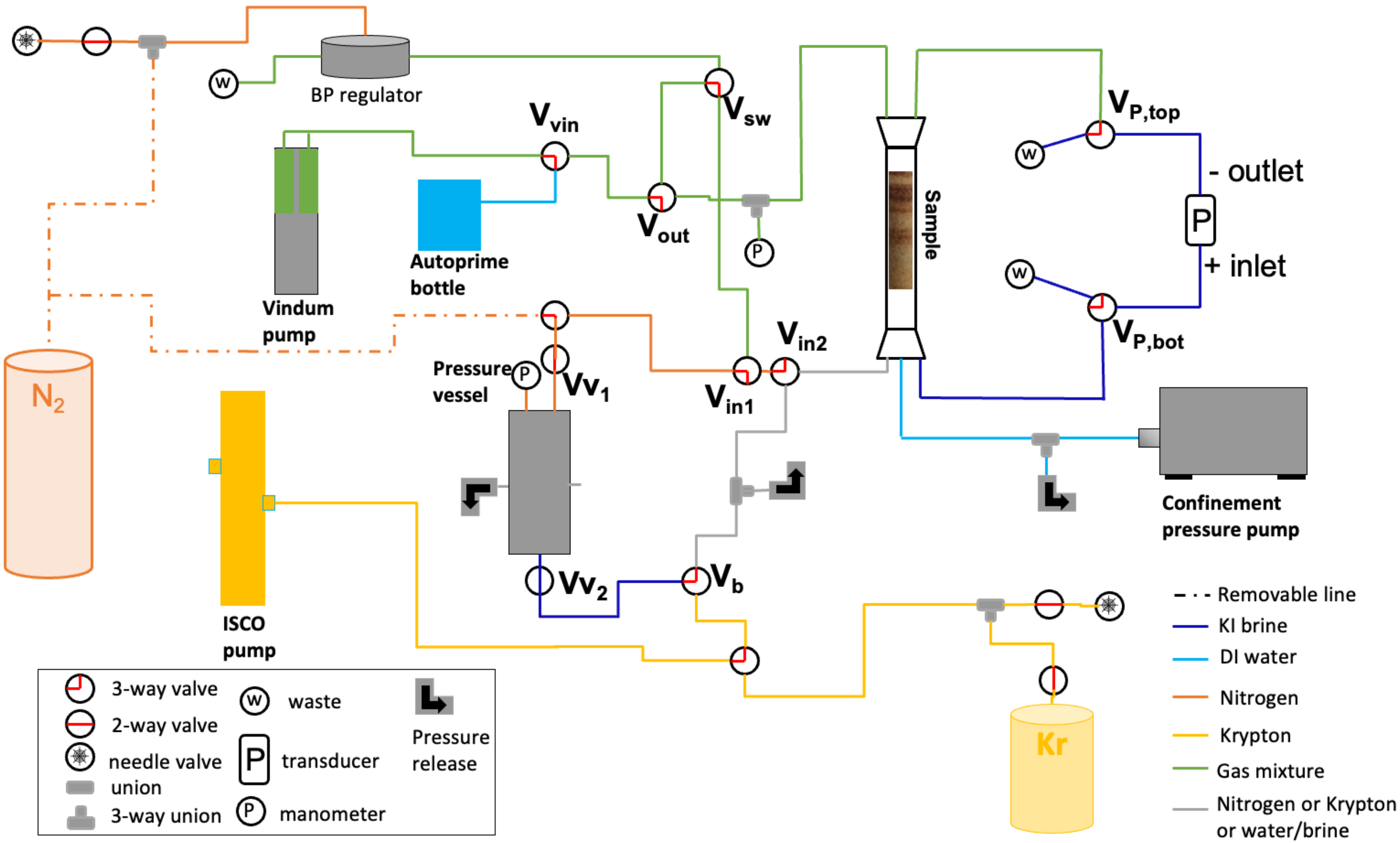


Fig. S1: Experimental setup diagram. The BP regulator was used primarily to bypass gas around the sample, without having to flow into the Vindum pump, as a purge control. The N2 gas bottle (connected with dashed lines) was used initially to fill the pressure vessel, and then as pressure control for the BP regulator.

## S2 Experimental conditions

Experimental conditions are shown in Fig. S2 below. Here, it is clear that the temperature, and pore pressure are stable throughout the experiments, with little variation between experimental runs. The pore pressure of the sample is maintained at ($\overline{P} = 3.57$ MPa, $\sigma(P) = 0.029$ MPa) and temperature ($\overline{T} = 22.6$ °C, $\sigma(T) = 0.17$ °C). The pressure drop highlights the clear impact of multiphase flow when nitrogen gas enters the sample in experiment PT and DG, initially displacing brine before mixing with the krypton. In experiment CG, the nitrogen mixes with the krypton straight away, with little brine displacement or change in saturation, so exhibits a stable pressure drop throughout the experiment. The spike in pressure around t = -1.2 hours is when the valves were switched to initiate nitrogen injection into the sample, with dead volume traversal then taking around 70-80 minutes as detailed above.

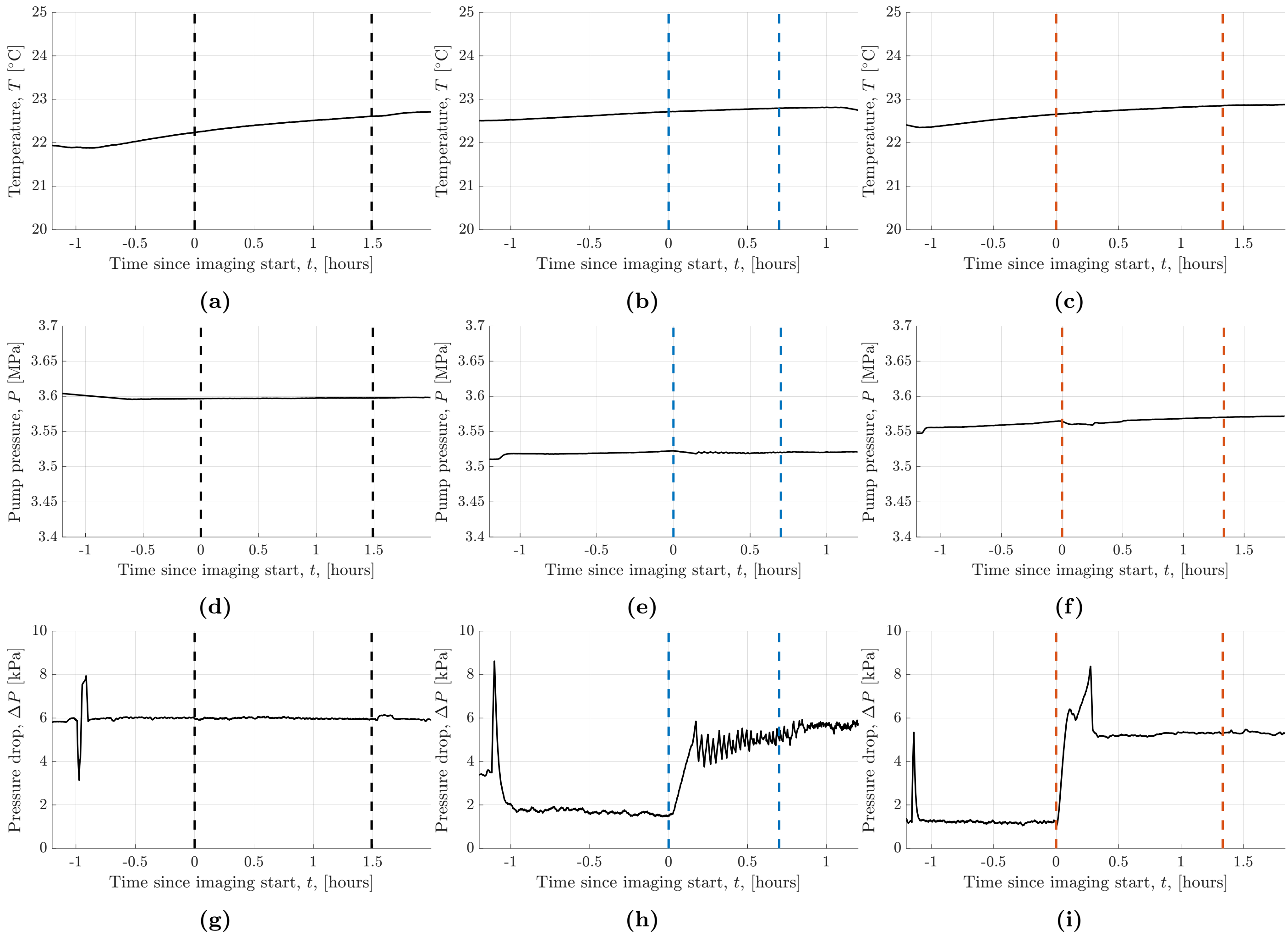


**Fig. S2: Experimental temperatures (first row), pump pressure (middle row) and pressure drop across the sample (last row), for experiment CG (first column), experiment PT (middle column) and experiment DG (last column). The time-period shown is 1.2 hours (72 minutes) before the scanning starts until 30 minutes after scanning ends, for each experiment. The dashed vertical lines indicate the scanning period. The temperature is measured by a transducer at the differential pressure sensor, which gives an indication of the average temperature of the sample and fluids. The pump pressure is the pressure measured by the Vindum pump, and is indicative of the pore pressure during the experiment, given the negligible differential pressure. The pressure drop is the difference in absolute pressure between the top and bottom of the sample, calibrated to remove hydrostatic effects.**

## S3 X-Ray CT scanning setup

X-Ray CT micro-CT imaging was performed with the Environmental Micro-CT (EMCT) scanner in the Centre for X-Ray Tomography (UGCT) at the University of Ghent. The EMCT scanner featured a rotating cone-beam source and detector, with a fixed sample, allowing fast, stable X-Ray CT scanning. See Fig. S3 below for an image of the rotating scanner setup and core-holder.

We used a scanning energy of 130 kV, a power of 16 W, a 20 ms exposure and 1001 projections for each tomographic scan. We used 2x averages for each projection, resulting in a 40 second acquisition time. The spatial resolution was 12.6 $\mu$m with a field-of-view of 8 x 8 x 8 mm. Due to the novel source-detector setup, the scanner could continuously rotate, acquiring projections sequentially.

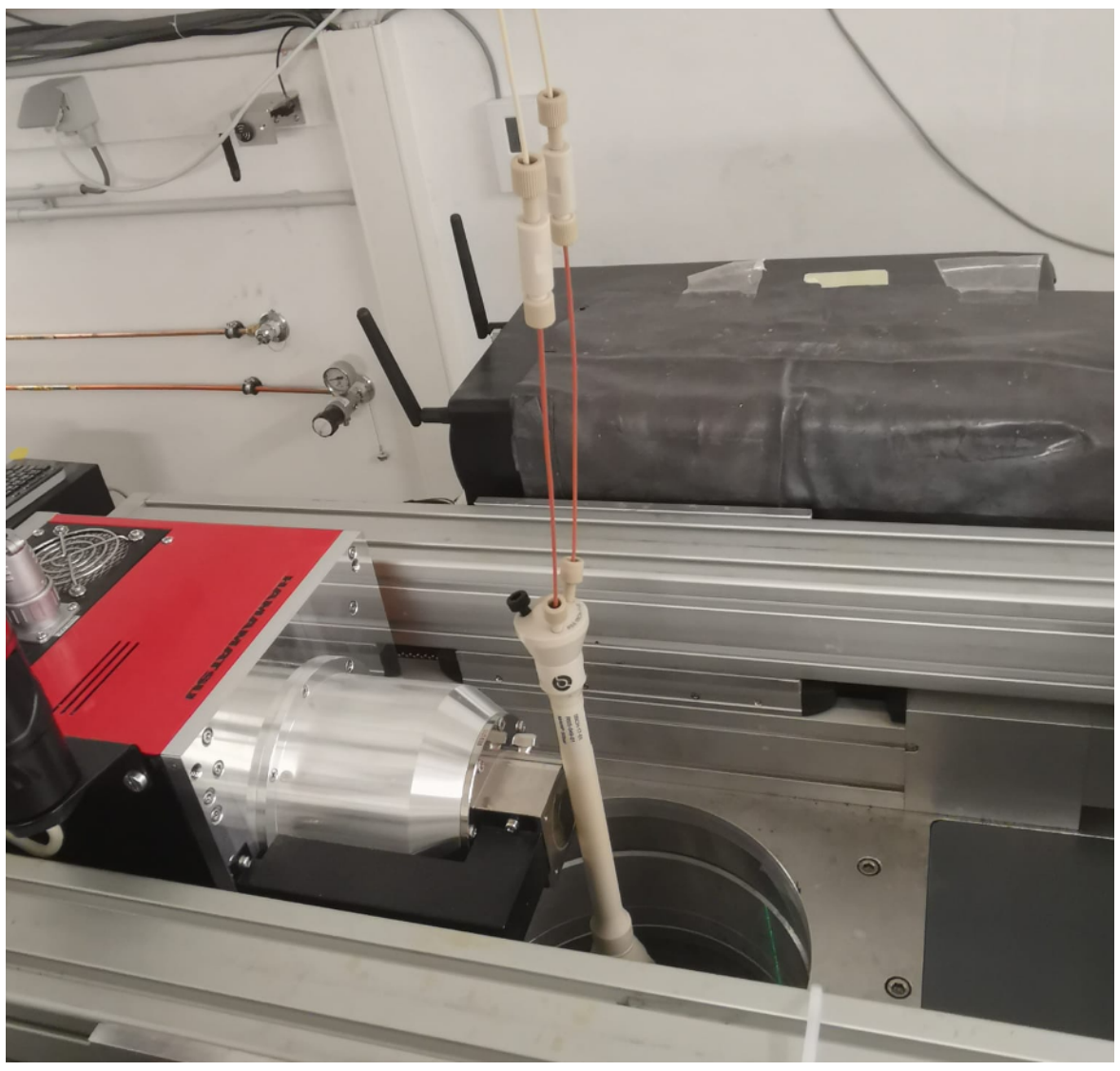

**Fig. S3: EMCT scanner and flow cell at UGCT, Ghent University. The flow cell is shown next to the rotating X-Ray source (left of the image in red). The cell has two outlet lines, one for flow to the Vindum receiving pump, and the other to one side of the pressure transducer. The rectangular metal mount and X-Ray source (red) rotate around the sample as images are acquired.**

## S4 Gas composition calibration

Krypton and nitrogen were chosen as analogues for natural gas and hydrogen, respectively, due to their comparable fluid flow behaviour, but high attenuation contrast under typical laboratory X-Ray micro-CT energies. Table S1 shows that at the given experimental conditions, krypton and nitrogen have distinct relative intensities through an idealised medium (pure material with a depth of 6 mm) with $I/I_0 = 0.70$ and 1.00, respectively, whereas methane and hydrogen are indistinguishable with $I/I_0 = 0.99$ and 1.00, respectively.

Calibration experiments were initially performed with the sample saturated with either krypton or air (as proxy for nitrogen, with near identical attenuation behaviour at the experimental conditions) at selected absolute pressures ranging from atmospheric (0.1 MPa) to 4 MPa. The greyscale intensity peak in the saturated pore space at the varying partial pressure of krypton is plotted in Fig. S4. It is clear that the krypton's attenuation is linearly proportional to pressure, with the linear fit coinciding with the air (and therefore nitrogen) attenuation at 0 krypton partial pressure.

Assuming ideal gas behaviour (both gases exhibit <8% variation in real gas density vs.

**Table S1: Fluid properties and relative X-Ray intensities ($I/I_0$) in different settings; field (reservoir), laboratory and reference. Mass attenuation coefficients are reported at an energy of 50 KeV. Water values are for the 20wt% KI brine used in the experiments. $I/I_0$ is calculated using Beer-Lambert's law with a path length of 6 mm.**

| Location | Reservoir | | Laboratory | | Reference | |
|---|---|---|---|---|---|---|
| Fluid | $H_2$ | $CH_4$ | $N_2$ | Kr | $H_2O$ | $SiO_2$ |
| Temperature [°C] | 68.0 | 68.0 | 22.6 | 22.6 | - | - |
| Pressure [Mpa] | 15.0 | 15.0 | 3.6 | 3.6 | - | - |
| Density [kg/m$^3$] | 9.85 | 93.73 | 41.21 | 132.76 | 1000 | 2650 |
| Viscosity [Pa.s] | 9.94x10$^{-6}$ | 1.61x10$^{-5}$ | 1.83x10$^{-5}$ | 2.67x10$^{-5}$ | 1x10$^{-3}$ | - |
| Density ratio with $H_2O$, $\rho_r$ [-] | 0.010 | 0.094 | 0.041 | 0.133 | 1 | - |
| Viscosity ratio with $H_2O$, $M_r$ [-] | 0.010 | 0.016 | 0.018 | 0.027 | 1 | - |
| Mass attn. coeff [cm/g] | 0.336 | 0.224 | 0.198 | 4.523 | 2.107 | 0.319 |
| Relative intensity $I/I_0$ [-] | 1.00 | 0.99 | 1.00 | 0.70 | 0.28 | 0.60 |

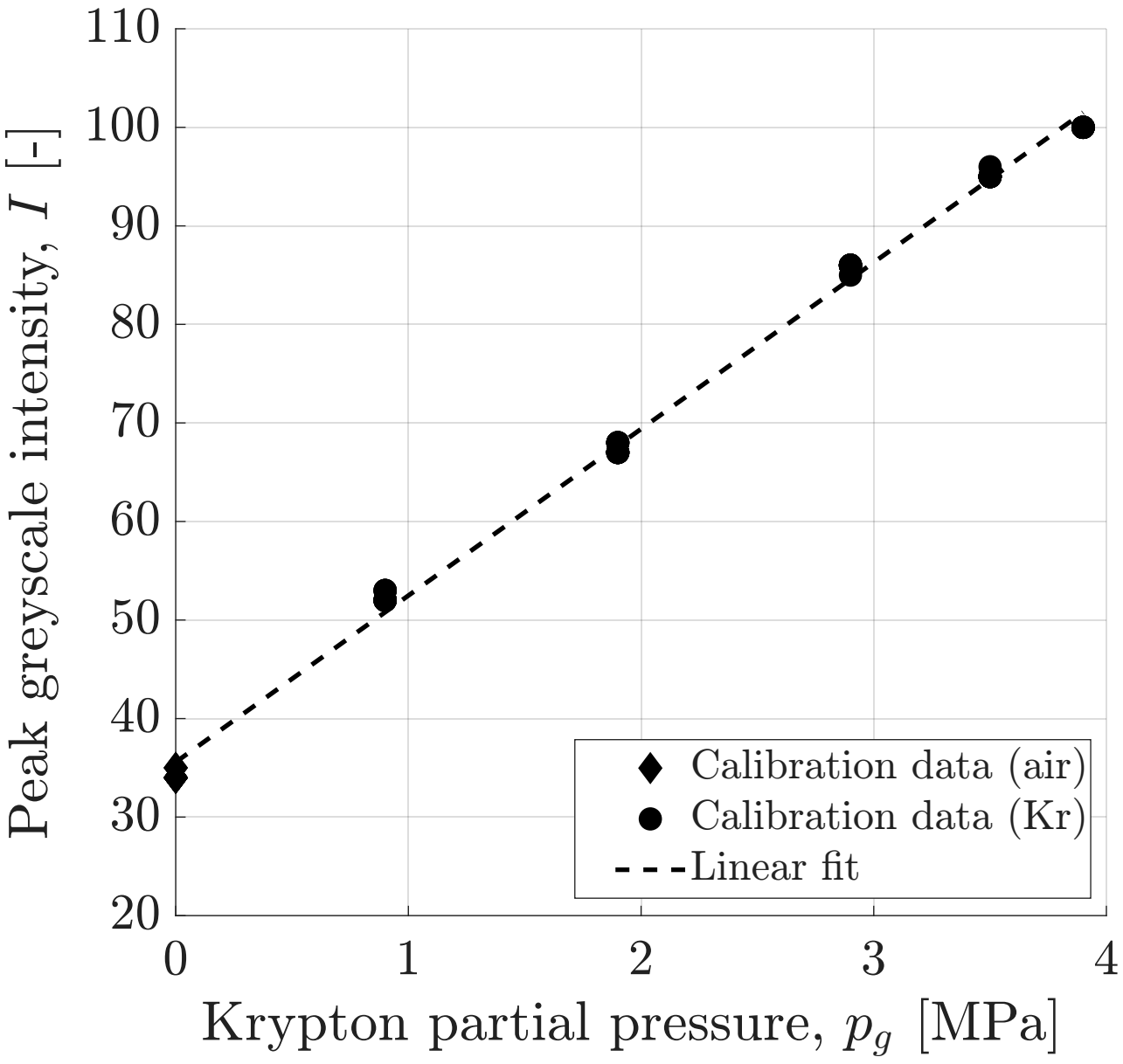


(a)

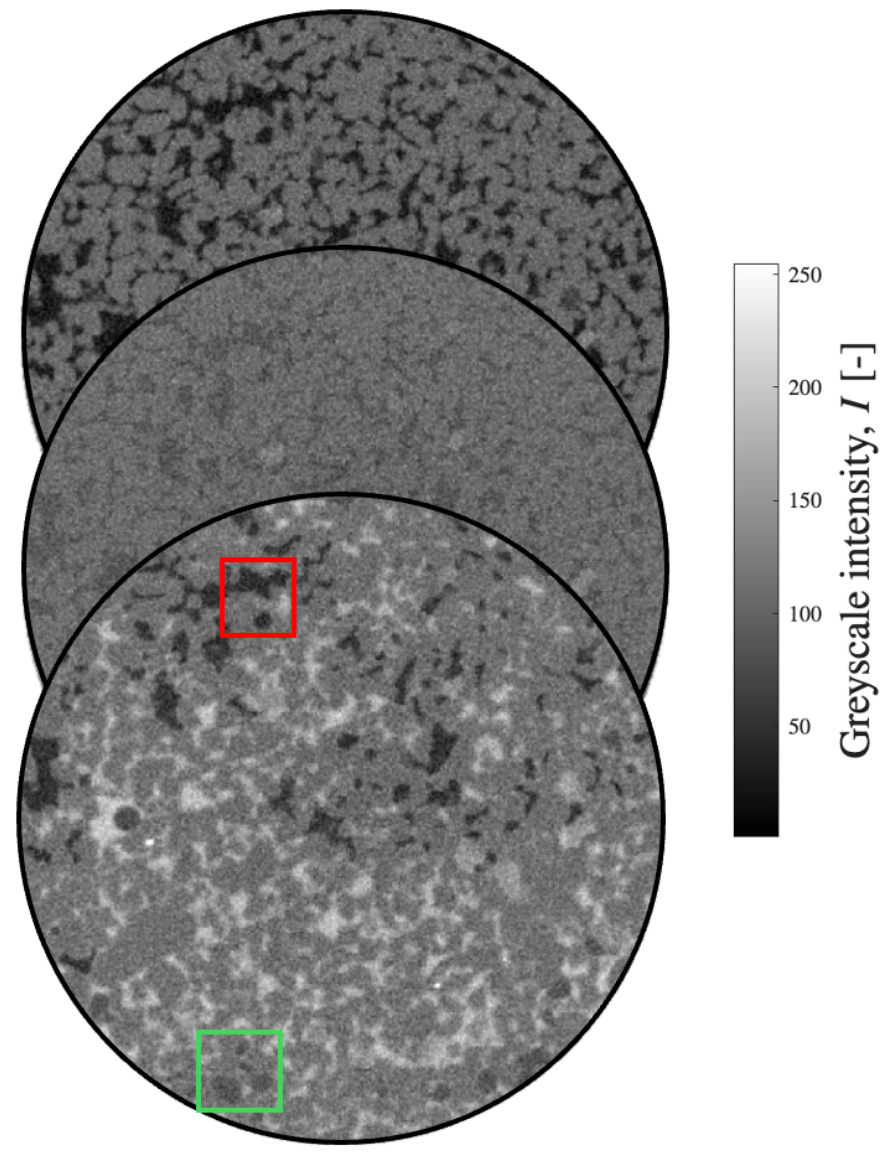


(b)

**Fig. S4: Calibration data for the experiments. (a) Peak greyscale intensity in the pore space as a function of krypton partial pressure. There are 12 distinct points (scans) at each pressure which overlap considerably. (b) Raw X-Ray micro-CT slices through the centre of the sample at 3 different conditions: top - air saturated at atmospheric conditions, middle - krypton saturated at experimental conditions (3.6 MPa), bottom - krypton/nitrogen and brine during the third experiment at the point of maximum variance. The phases in order of ascending greyscale are nitrogen, krypton, rock and brine. The green box shows gas near 100% krypton composition, the red box shows gas near 100% nitrogen composition.**

ideal gas density over the experimental conditions) and near-constant experimental pressure ($\overline{P}$ = 3.57 MPa, $\sigma(P)$ = 0.029 MPa) and temperature ($\overline{T}$ = 22.6 °C, $\sigma(T)$ = 0.17 °C) the voxel-wise gas composition, $c$ in the pore space can therefore be calculated from the

calibration images using a linear mapping:

$$c = \frac{I_{kr=1} - I_{exp}}{I_{kr=1} - I_{n2=1}} \tag{S1}$$

where $I_{kr=1}$, $I_{n2=1}$ and $I_{exp}$ are the reconstructed greyscale intensities for the krypton calibration image, nitrogen (or air) calibration image and the experimental image, respectively.

## S5 Image processing

Each batch of 1001 projects (taking in total 40 s) were reconstructed using the Panthera software from Tescan, with automated centre of rotation retrieval. After reconstruction, images were initially processed using the following steps:

1. Images were first registered to a high-resolution (6 $\mu$m) dry-scan image of the pore space. This was performed in Avizo using normalized mutual information, and mainly features small rotational corrections to account for the small-drift over many rotations.

2. Images were re-sampled to a consistent grid using the Lanczos algorithm in Avizo, followed by masking and cropped to remove the sleeve and regions impacted by the cone-beam geometry. The resulting images are 436 x 436 x 560 pixels, with 560 vertical pixels covering the sample.

3. Images are converted to 8 bit in Avizo using a linear mapping, with scaling of 0.006375 and offset of -15000, with a final range of 0-255.

4. Images were filtered in Matlab using the ImageProcessing toolbox. A non-local means filter, with a search window size 15, comparison window size 5, smoothing level 15.

An example raw and filtered image generated by this process are shown in Fig. S5a, b, respectively. Following this initial post-processing, the ImageProcessing toolbox in Matlab was used to generate final saturation and gas composition images. Firstly, the dry image (air saturated) was threshold segmented with a greyscale value of 70 to generate a binary pore space image. Small-cluster removal was performed using the connected components *bwconncomp* function, with clusters $<$9 voxels removed. Two pore space images were then generated, one in which the pore space is eroded by 1 voxel using the *imerode* function with a cubic morphological element, and the other which is left unaltered. Both pore space images are then modified to have pore = 1, everything else = NaN. These are used in later processing of saturation and gas composition images.

For the saturation images, gas is segmented from the filtered images using a greyscale value of 120 to generate a binary gas phase image. We then apply the same small cluster removal as detailed above. The image is then multiplied by the unaltered pore space image, to give gas = 1, brine = 0, and rock = NaN, to allow efficient calculations of means omitting NaNs.

For composition images, we use the experimental image in question with the calibration images to generate a voxel-wise gas composition map, $c$ in the pore space, using Eq. (S1) above. This generates a gas composition at every single pore and grain voxel. We then multiply the gas composition image by the corresponding segmented gas phase image (gas = 1, everything else = NaN), to produce a gas composition map only inside the gas phase (everything else has NaN). We then multiply this image by the eroded pore space image (pore = 1, everything else = NaN) to remove partial volume effects from the fluid-grain contacts, which can lead to erroneous gas compositions. Finally small clusters are removed from the image as per the previous algorithm.

During the experiments, it is possible that pressure variations could impact the fluid density and hence attenuation, which could be wrongly attributed to gas composition changes. The value of $dc/dp_g = 0.286$ MPa$^{-1}$ from Fig. S4 (when the greyscale intensity range of 34 to 96 across the partial pressure range is converted to the composition range of 0 to 1) means that the experimental pressure variance of $\Delta P = 0.029$ MPa would only result in a composition change of $\Delta c = 0.0083$. Similarly, capillary pressure variations result in negligible changes in attenuation. The minimum (permitted in the image after processing) trapped cluster size of

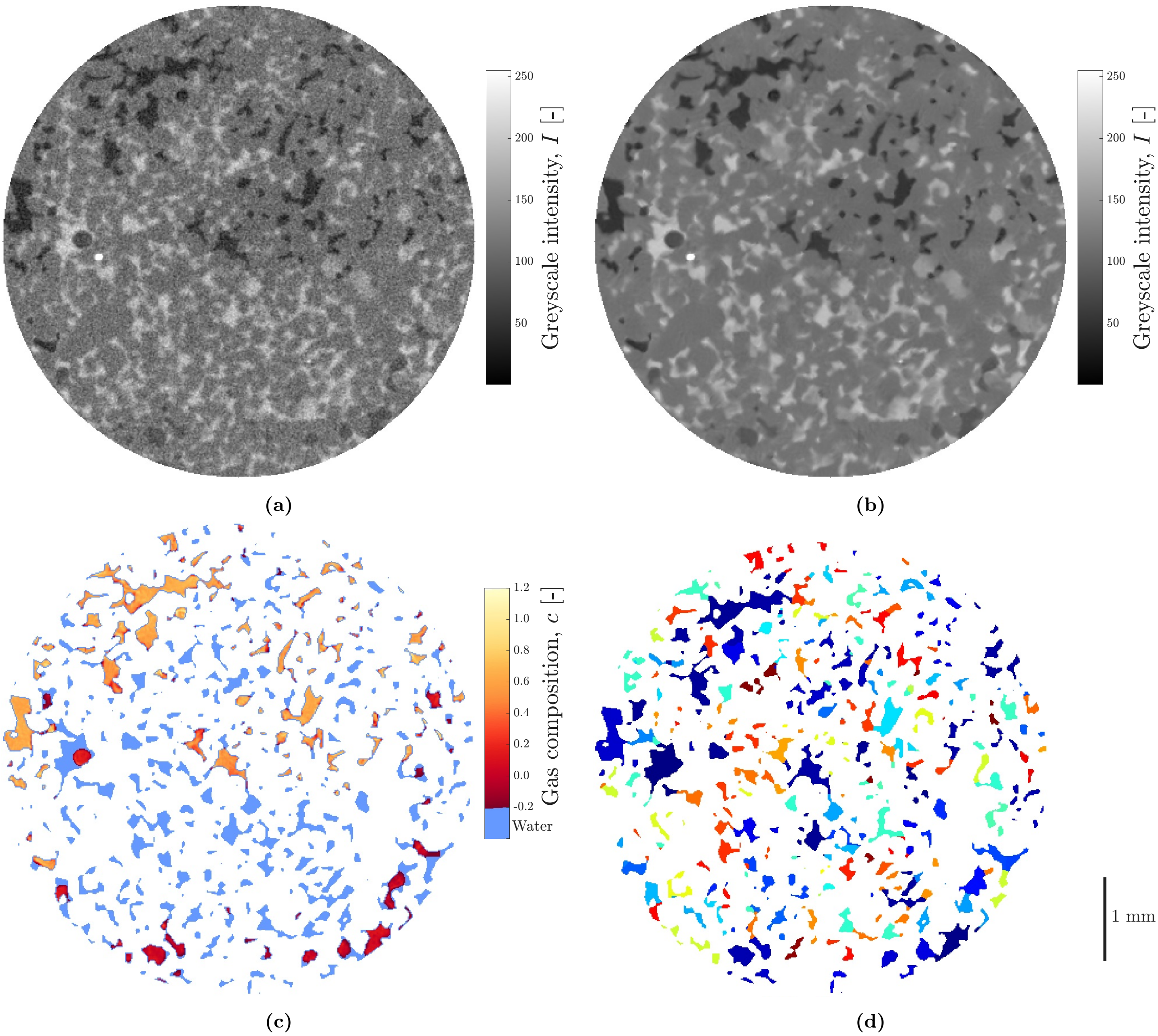


**Fig. S5: Image processing workflow showing a 436 x 436 pixel slice through the centre of the imaged region for experiment DG, at scan number 25 (point of maximum variance in the main text). (a) Raw image after registration, cropping and conversion to 8 bit. (b) Filtered image after non-local means filtering. (c) Gas composition image after differential calculation with the calibration images. (d) Pore body image of the pore space using the pore network extraction algorithm of [1]. Each segmented pore body has a different RGB colour, chosen randomly. Note, in (c) and (d), the rock phase is transparent. The scale bar for all images is shown in the bottom left of (d).**

1 voxel radius, 12.6$\mu$m, would have a capillary pressure $P_c \approx 11$ kPa, which would impact composition interpretation by only $\Delta c = 0.003$.

The gas composition images are used to calculate gas composition means and variance in the main paper. The gas composition mean presented in the main paper is the mean across the entire image. The gas composition variance is the variance of the 1D gas composition mean through the vertical z-axis of the sample, with a spatial support of 100 $\mu$m. We use the variance in the 1D mean, as opposed to 2D or 3D variance to allow direct comparison with the 1D simulations, and to minimise noise at the local voxel level.

The saturation images are used to calculate saturation means in the main paper. Due to the fast, continuous scanning, it is possible that motion artefacts appear as intermediate greyscale when a pore changes from water to gas (krypton or nitrogen) during the scanning period, or vice-versa. While this does occur infrequently, it does not impact the results as the intermediate phase only lasts for a single time-step and generally produce erroneous segmentations which can be readily removed from statistical analyses of pore-filling etc.,

The saturation images are also used as the basis for Euler characteristic calculations, along with the unaltered pore space image. We use these images, as opposed to the gas composition image with eroded pore space to have a more representative estimate of connectivity of the gas phase. The Euler characteristic is calculated using the *imEuler3D* function from the *imMinkowski* package, using a 3D connectivity of 26 [2]. We normalise the Euler characteristic, $\chi_g$ against the dry pore space Euler characteristic $\chi_p$, to produce a positive normalised Euler characteristic.

The unaltered pore space image is used to generate the pore network model. The pore space is divided into pore bodies using the algorithm of [1], generating 2901 bodies and 8276 connecting pore throats. The image based voxelised gas compositions are mapped onto each pore body to generate averaged pore body gas compositions in time. From this, a 4 parameter logistic function is fitted to the data:

$$c_b = x(4) + \frac{x(1) - x(4)}{(1 + t/x(3))^{x(2)}} \tag{S2}$$

where $c_b$ is the pore body average gas composition, $t$ is time, and $x(1), x(2), x(3)$ and $x(4)$ are the fitting parameters representing the minimum gas composition, slope, inflection point and maximum gas composition, respectively. The model is fitted to the gas composition-time data using the *fmincon* function in Matlab, which minimises the RMSE between model and data using an interior-point algorithm.

## S6 1D Gas composition and saturation evolution

We show the 1D gas compositions and liquid saturations across the three experiments in Fig. S6 below. The first 10 time-steps are shown before nitrogen enters the sample, with near constant conditions: $c_z \approx 0$ in all experiments, and $S_l \approx 0.2, 0.5$ and $0.8$ in experiments CG, PT and DG, respectively. We note that there is frontal advance behaviour in experiment DG for approximately 8 time steps after nitrogen hits the sample inlet. After these 8 time steps nitrogen has percolated across the image domain. It takes approximately 14 further time steps for the saturation to reach near steady-state.

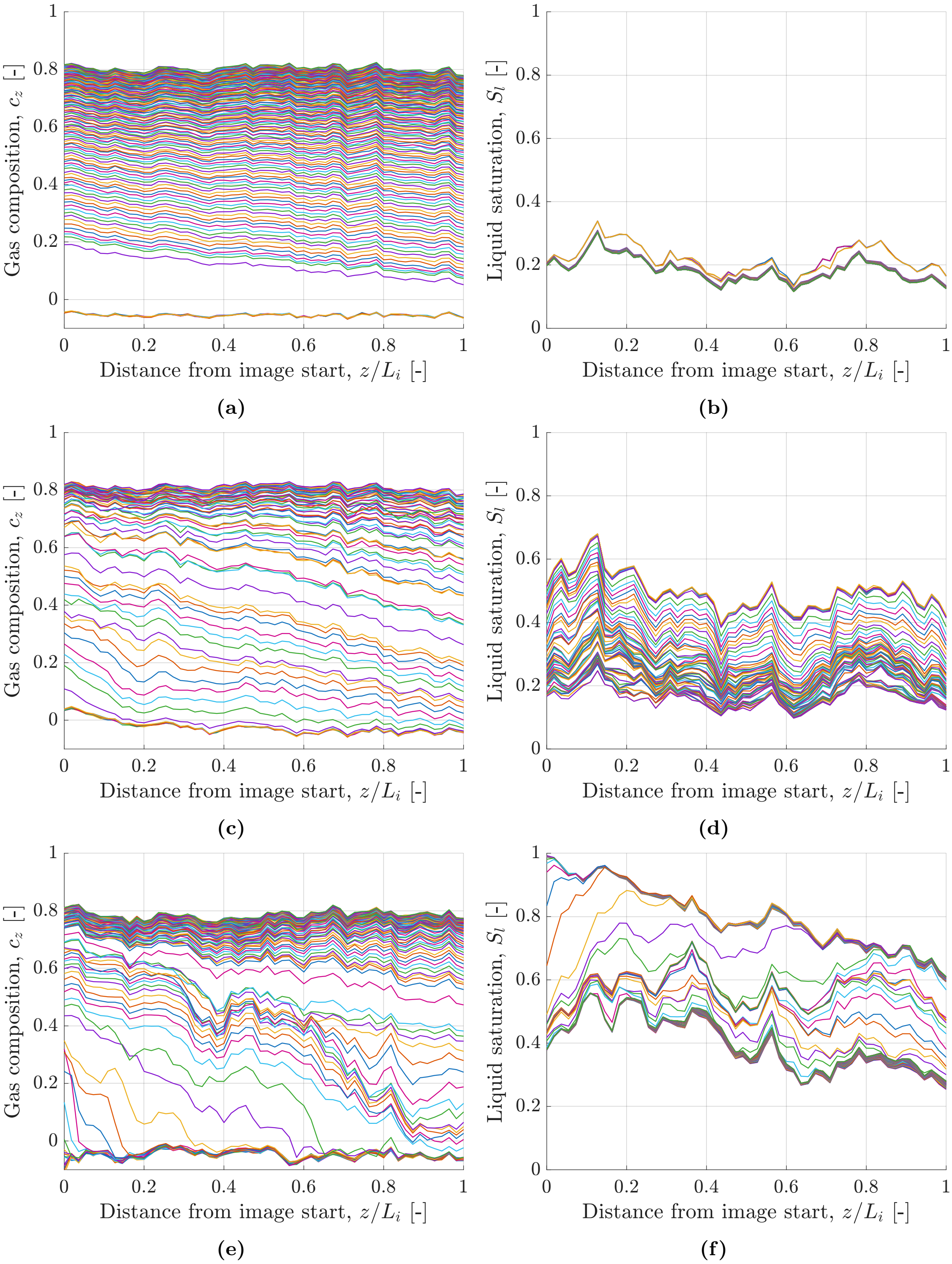


**Fig. S6: Longitudinal 1D mean gas compositions (left hand column) and liquid saturations (right hand column) for the three experiments (rows 1 to 3 for experiments CG, PT and DG, respectively). The averaging support scale is 100 $\mu$m. Colours represent discrete image time-steps. We show 145, 74 and 131 distinct time-steps across experiments CG, PT and DG, respectively, with the first 10 time-steps showing results before nitrogen entered the sample. Multiple lines can be superimposed on each other during steady-state behaviour. Gas compositions are increasing in time, liquid saturations are decreasing in time.**

# S7 1D modelling based on the Advection Diffusion Equation (ADE)

We utilise a classical 1D advection-diffusion equation, with multi-zone formulation to capture the gas composition transport in the tubing and sample flow, given a constant porosity and saturation in each zone. The PDE for each zone $i$ (tubing or sample) is given by:

$$\frac{\partial c_z}{\partial t} = D_i \frac{\partial^2 c_z}{\partial z^2} - \frac{Q_i}{A_i \phi_i S_g} \frac{\partial c_z}{\partial z} \tag{S3}$$

where $c_z$ is the vertical, z-axis longitudinal gas composition, $t$ is time, $D_i$ is the diffusion coefficient, $Q$ is the volumetric flux ($Q_i = u_i A_i \phi_i S_{gi}$), $A_i$ is the cross-sectional area, $\phi_i$ is the porosity, $q = u_i \phi_i S_{gi}$ is the Darcy velocity, $u_i$ is the pore scale velocity and $S_{gi}$ is the gas saturation, $S_{gi} = 1 - S_{li}$. Note, this assumes a constant, immobile gas saturation. The interface conditions between the tubing and inlet are continuity of gas composition and flux:

$$c_1 = c_2, \qquad A_1 \phi_1 S_{g1} D_1 \frac{\partial c_z}{\partial z} = A_2 \phi_2 S_{g2} D_2 \frac{\partial c_z}{\partial z} \tag{S4}$$

where we have enforced continuity of the projected volumetric flux. At the boundaries of the domain (tubing inlet, $z = 0$) and sample outlet ($z = L$) we use a Dirichlet and Neumann condition, respectively:

$$c_z(0, t) = c_v, \qquad \frac{\partial c_z}{\partial z}|_{z=L_t} = 0 \tag{S5}$$

where $c_v$ is pressure vessel gas composition (connected to the tubing). For initial conditions, we either specify $c_z(0 \leq z \leq L) = 0$ (Sim 1) or $c_z(0 \leq z \leq L_t) = c_v$ and $c_z(L_t < z \leq L) = 0$ (Sim 2) where $L_t$ is the tubing length. For the tubing flow, the diffusion coefficient is equal to the bulk diffusion coefficient between nitrogen and krypton, i.e. $D_1 = D_b$ and $\phi = 1$. For the sample flow, the diffusion coefficient is set as $D_2 = D_b \tau$ where $\tau$ is the porous media tortuosity, approximated here by a Bruggeman type formulation, $\tau = \phi^{1/2} S_g^{1/2}$. The effective, continuum diffusivity coefficient in the sample is then given by $D_{eff} = \phi \tau S_g D_b$ [3].

This formulation allows the advection & diffusion of nitrogen from the pressure vessel (constant gas composition boundary $c_v \approx 1$), through the tubing and into the sample. The simulation has four parameters with uncertainty: the tubing length, $L_t = 42$ cm, sample porosity $\phi = 0.24$ (higher than image porosity to account for sub-resolution porosity), bulk diffusion coefficient of nitrogen-krypton, $D_b = 3 \times 10^{-7}$ m$^2$/s (in-line with known noble gas diffusion coefficients at atmospheric conditions and ideal gas pressure scaling [4, 5, 6]) and the pressure vessel gas composition, $c_v = 0.85$ (necessarily $< 1$ due to back-diffusion of krypton from previous calibration experiments). We take the gas saturation as $S_g = 0.8$, and vary it $\pm 0.2$ to account for variations between the experiments. These parameters are initially estimated from experimental measures and literature values, then refined through an ensemble sweep across the likely ranges, with the lowest $L_2$ error between simulation and experimental gas composition profiles determining the final values. The resulting parameters are non-unique, but can only vary within $\approx 20\%$ to achieve similar convergence to the experimental results.

Eq. (S3) - (S5) are discretised using a central difference approach for spatial gradients. Two 'ghost nodes' are used either side of the interface, which are infinitesimally close to the interface to enforce interface conditions, without requiring a node explicitly at the interface. The finite difference equations are solved temporally using a direct, fully implicit scheme. A time-step size of 10 s and spatial discretisation of 0.2 mm are used, which result in mesh and time-independent numerical solutions. The result of the ADE simulation for experiment CG is shown in Fig. S7.

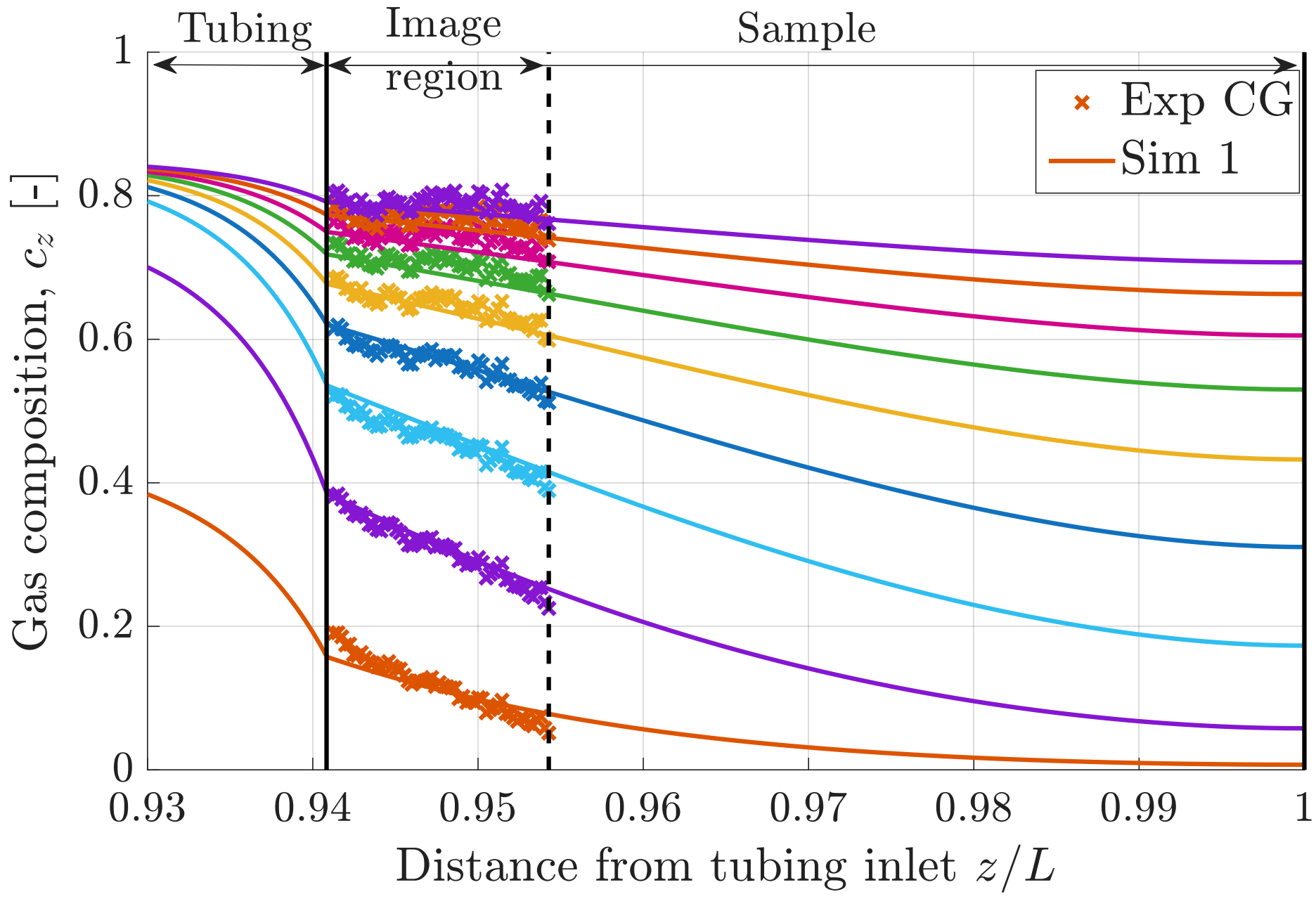


**Fig. S7: Longitudinal 1D mean gas compositions in the experiment vs. simulation through time. The length is non-dimensionalised against the whole system length $L$ with the imaging region highlighted. Note, simulation results span the entire tubing and sample region, whereas the experimental imaging region only covers the first quarter of the sample. Results are shown at 10 minute increments, with the first result at t = 10.9 minutes ($t' = 62.5$). The $C^0$ continuity at the tubing-sample interface is due to the 1D averaging of the 3D gas composition in zones with vastly different cross-sectional areas, i.e. the tubing diameter = 0.075 mm whereas the sample diameter = 6 mm.**

## S8 Supporting video legends

1. Video S1. 2D rendering of the gas composition and gas-water distribution during experiment CG. We show an x-z cross-section taken through the centre of the injection port. Krypton gas ($c = 0$), nitrogen ($c = 1$), water (blue), rock (dark grey) and the PEEK inlet (light grey) are shown.

2. Video S2. 2D rendering of the gas composition and gas-water distribution during experiment PT. We show an x-z cross-section taken through the centre of the injection port. Krypton gas ($c = 0$), nitrogen ($c = 1$), water (blue), rock (dark grey) and the PEEK inlet (light grey) are shown.

3. Video S3. 2D rendering of the gas composition and gas-water distribution during experiment DG. We show an x-z cross-section taken through the centre of the injection port. Krypton gas ($c = 0$), nitrogen ($c = 1$), water (blue), rock (dark grey) and the PEEK inlet (light grey) are shown.

4. Video S4. 3D rendering of the gas composition during experiment CG. We show a cutout of the full cylindrical sample taken through the centre of the injection port. Krypton gas ($c = 0$) and nitrogen ($c = 1$) are shown, the rock and water phases are made transparent.

5. Video S5. 3D rendering of the gas composition during experiment PT. We show a cutout of the full cylindrical sample taken through the centre of the injection port. Krypton gas ($c = 0$) and nitrogen ($c = 1$) are shown, the rock and water phases are made transparent.

6. Video S6. 3D rendering of the gas composition during experiment DG. We show a cutout of the full cylindrical sample taken through the centre of the injection port. Krypton gas ($c = 0$) and nitrogen ($c = 1$) are shown, the rock and water phases are made transparent.

7. Video S7. 2D rendering of the gas composition and gas-water distribution during experiment CG. We show a zoomed in view around 'Pore 1', described in the main text. Krypton gas ($c = 0$), nitrogen ($c = 1$), water (blue) and rock (white) are shown.

8. Video S8. 2D rendering of the gas composition and gas-water distribution during experiment PT. We show a zoomed in view around 'Pore 1', described in the main text. Krypton gas ($c = 0$), nitrogen ($c = 1$), water (blue) and rock (white) are shown.

9. Video S9. 2D rendering of the gas composition and gas-water distribution during experiment DG. We show a zoomed in view around 'Pore 1', described in the main text. Krypton gas ($c = 0$), nitrogen ($c = 1$), water (blue) and rock (white) are shown.

10. Video S10. 2D rendering of the gas composition and gas-water distribution during experiment CG. We show a zoomed in view around 'Pore 2', described in the main text. Krypton gas ($c = 0$), nitrogen ($c = 1$), water (blue) and rock (white) are shown.

11. Video S11. 2D rendering of the gas composition and gas-water distribution during experiment PT. We show a zoomed in view around 'Pore 2', described in the main text. Krypton gas ($c = 0$), nitrogen ($c = 1$), water (blue) and rock (white) are shown.

12. Video S12. 2D rendering of the gas composition and gas-water distribution during experiment DG. We show a zoomed in view around 'Pore 2', described in the main text. Krypton gas ($c = 0$), nitrogen ($c = 1$), water (blue) and rock (white) are shown.

## References


[1] H. Dong and M. J. Blunt. "Pore-network extraction from micro-computerized-tomography images". In: *Physical Review E* 80.3 (2009). DOI: 10.1103/physreve.80.036307.

[2] D. Legland. *imMinkowski,* 2025. URL: https://au.mathworks.com/matlabcentral/fileexchange/33690-imminkowski.

[3] R. Helmig. *Multiphase flow and transport processes in the subsurface. A contribution to the modeling of hydrosysteme*. Environmental engineering. Berlin: Springer, 1997. 367 pp. ISBN: 9783642645457.

[4] W. Hogervorst. "Diffusion coefficients of noble-gas mixtures between 300 K and 1400 K". In: *Physica* 51.1 (1971), pp. 59–76. DOI: 10.1016/0031-8914(71)90137-6.

[5] T. W. Patzek, D. B. Silin, S. M. Benson, and G. I. Barenblatt. "On Vertical Diffusion of Gases in a Horizontal Reservoir". In: *Transport in Porous Media* 51.2 (2003), pp. 141–156. DOI: 10.1023/a:1021957416536.

[6] S. Kobeissi, N. N. Ling, E. F. May, and M. L. Johns. "Measurements of intra-diffusion coefficients for gaseous binary mixtures". In: *Chemical Engineering Science* 303 (2025), p. 120952. DOI: 10.1016/j.ces.2024.120952.
